# Prediction of key biological processes from intercellular DNA damage differences through model-based fitting

**DOI:** 10.1016/j.isci.2024.111473

**Published:** 2024-11-26

**Authors:** Kensuke Otsuka, Kouki Uchinomiya, Yuki Yaguchi, Atsushi Shibata

**Affiliations:** 1 Biology and Environmental Chemistry Division, Sustainable System Research Laboratory, Central Research Institute of Electric Power Industry, 1646 Abiko, Abiko-shi, Chiba 270-1194, Japan; 2 Division of Molecular Oncological Pharmacy, Faculty of Pharmacy, Keio University, 1-5-30, Shibakoen, Minato-ku, Tokyo 105-8512, Japan

**Keywords:** Molecular biology, Mathematical biosciences, Cancer

## Abstract

DNA double-strand breaks (DSBs) occurring within the genomic DNA of mammalian cells significantly impact cell survival, depending upon their repair capacity. This study presents a mathematical model to fit fibroblast survival rates with a sequence-specific DSB burden induced by the restriction enzyme AsiSI. When cells had a sporadic DSB burden under mixed culture, cell growth showed a good fit to the Lotka–Volterra competitive equation, predicting the presence of modifying factors acting as competitive cell-to-cell interactions compared to monocultures. Under the predicted condition, we found the Acta2 gene, a known marker of cancer-associated fibroblasts, played a role in competitive interactions between cells with different DSB burdens. These data suggest that the progression to the cancer microenvironment is determined by genomic stress, providing clues for estimating cancer risk by reconsidering the fitness of cells in their microenvironment.

## Highlights

• Automated liquid handling allowed for quantitative cell mixing and drug treatment
• Cell growth under genomic stress aligns with the Lotka–Volterra competition model
• Consecutive 10 DSB yield activates *Acta2* expression in fibroblasts
• Data-driven-based modeling highlights unbiased biological pathways

## Graphical abstract

**Figure:**
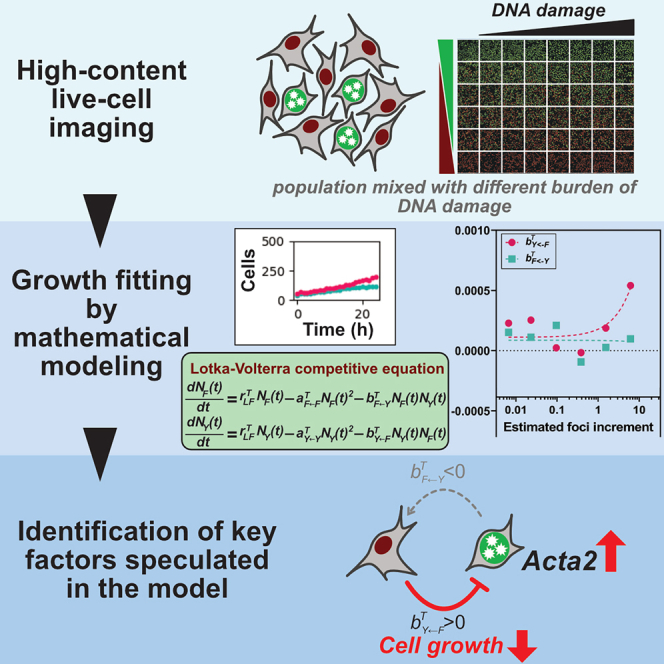

## Introduction

DNA double-strand breaks (DSBs) are critical events in mammalian cells intricately linked to subsequent cell survival.^1^ Exogenous factors, such as ionizing radiation, chemicals, and other environmental agents, can induce genomic stress in addition to endogenous factors. DNA repair mechanisms within cells are essential for preserving cellular health. However, the stable fixation of mutations associated with DNA misrepair, followed by abnormal gene expression and epigenetic changes, may initiate carcinogenic processes.^2^

When DSBs occur in proliferating cells, they typically undergo cell-cycle arrest to facilitate immediate repair of the cell damage. This growth arrest at the cell population level leads to a decrease in proliferative capacity. In cases of severe cell damage that cannot be repaired, mechanisms like apoptosis may be activated, resulting in programmed cell death.^3^ However, cells that survive despite being damaged or misrepaired may develop dysplasia, potentially giving rise to proto-oncogenic clones within the cell population. Understanding the fate of cells following DSB induction and their associated cell-cycle states is crucial for assessing tissue health and predicting disease emergence. Therefore, this study proposes four key issues to consider quantitatively when making such determinations and predictions: first, quantifying the threshold of DSBs that leads to cell death or survival; second, assessing the potential stabilization and expansion of surviving aberrant cells within the cell population; third, estimating the probability of dysplasia development over time; and finally, recognizing the promotion of dysplastic cells alongside quantitative changes in the tissue microenvironment. When unraveling the carcinogenic process at the tissue level, detecting clones with driver mutations in normal tissues^4^^,^^5^ suggests that the tissue microenvironment contributes to the malignancy of the mutant clones. The tumor microenvironment consists of stromal cells, immunocompetent cells, and blood vessels in the extracellular matrix (ECM).^6^ Fibroblasts, which make up the ECM, produce collagen and growth factors. One specific role of fibroblasts that is being investigated is their interactions with cancerous cells as cancer-associated fibroblasts (CAFs) in the tumor microenvironment.^7^ Recent findings suggest that the thickness of collagen in the ECM affects the progression of cancer clones.^8^ However, the precise alterations in fibroblasts that drive cancer progression remain elusive. This study hypothesized that it could be possible to predict quantitative changes in the microenvironment based on the number of DSBs and the cell-cycle state of cells, which reflect the properties and composition of their cell populations. To test this hypothesis, an *in vitro* approach using fibroblasts as a representative model of the tissue microenvironment was developed.

Evidence obtained from biological experiments is important for interpreting mechanisms under the same experimental conditions. However, inductive interpolation or extrapolation of missing data is challenging in terms of the quantitative prediction of basic phenomena. To address these challenges, mathematical modeling has been employed in various research fields, including machine learning^9^ and geostatistics.^10^ Mathematical models have also been utilized in oncology to understand the process of cancer progression^11^^,^^12^ and the interactions between cancer cells and their microenvironment.^13^

Various mathematical models have been developed in the field of population genetics to elucidate the dynamics of proliferating individuals. In a population consisting of multiple species, the dynamics of proliferation, which involve interactions among individuals, are explained by either the replicator dynamics or the Lotka–Volterra competition equation.^14^

This study focused on genome stress within fibroblasts, which form a crucial part of the tissue microenvironment. It investigated how the mixed composition of these fibroblasts changes under various genomic stress conditions and explored their interactions using live-cell imaging. Additionally, it analyzed the growth rate by applying evolutionary game theory. Mathematical models uncovering cell-to-cell interactions can be linked to investigating the correlation between gene expression and the DNA damage response level. This correlation aids in predicting the fate of the microenvironment under genomic stress. Such an approach proves valuable in determining the timing and extent to which damaged fibroblasts may serve as a scaffold for cancer development.

## Results

### Dose-dependent DSB burden visualized quantitatively through live-cell imaging

**Figure 1:**
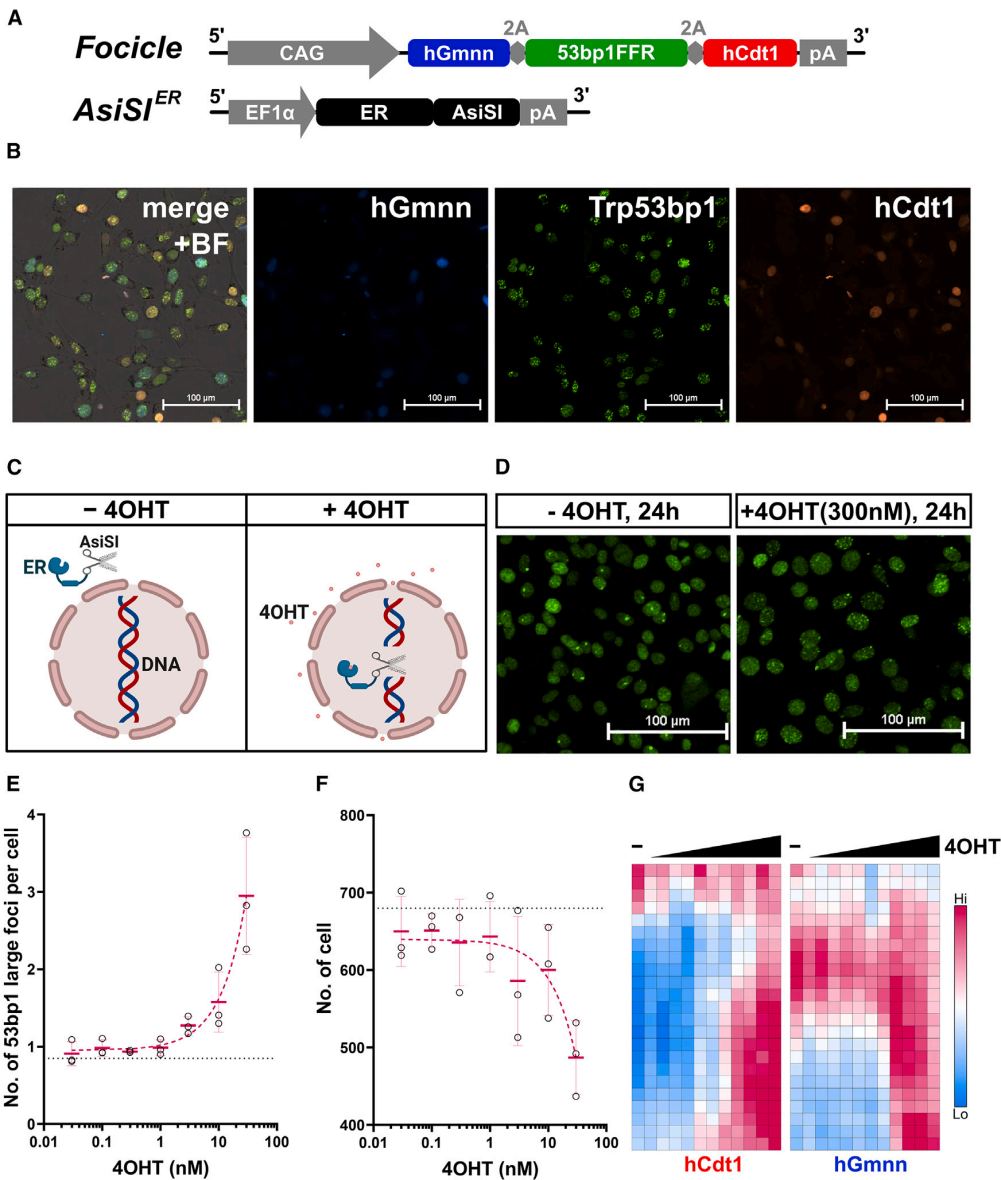
Experimental design for live-cell imaging of DSB repair and cell-cycle status in fibroblasts (A) Focicle cassette knocked in the ROSA26 region using CRISPR-Cas9 tricistronically expresses Trp53bp1 foci-forming region (FFR)/YFP, hGmnn/BFP, and hCdt1/RFP. Furthermore, a plasmid expressing the AsiSI/estrogen receptor (ER) was transfected into cells with piggyBac transposase. (B) Images of BFP, YFP, RFP, and merged images, including bright-field (BF) channels of Focicle-expressing NIH3T3 cells, 1 h after irradiation with 1 Gy X-rays. Scale bars, 100 μm. (C) Addition of tamoxifen (4OHT) to the medium-induced DSBs by AsiSI, along with the nuclear translocation of ER. Illustrations were created with Biorender.com. (D) Images of the YFP channel 24 h after adding 300 nM 4OHT or in the absence of 4OHT. The addition of 4OHT caused numerous foci of DSBs in the nuclei. Scale bars, 100 μm. (E) The number of cells in the microscopic field of view 20 h after the addition of various concentrations of 4OHT. The mean values are indicated by red dashes, and the dotted lines represent the mean values in the absence of 4OHT in triplicate in one representative experiment. Solid magenta lines and error bars indicate their mean ± SD. Dotted line indicates the median in the absence of 4OHT treatment. (F) Mean number of 53bp1 large foci per cell at 20 h after adding various concentrations of 4OHT. Symbols are identical to those shown in (E) in triplicate in one representative experiment. Solid magenta lines and error bars indicate their mean ± SD. Dotted line indicates the median in the absence of 4OHT treatment. (G) Heatmap of 4OHT concentration versus the time course of nuclear hCdt1 or hGmnn fluorescence intensity every 1 h from top to bottom.

We previously established a live-cell assay to quantitatively observe cell growth in real time and monitor the formation and repair of DSBs. We use the term "Focicle" to describe DSB foci and their relationship to the cell cycle. This technique enables fluorescent live-cell imaging of DSB repair proteins and cell-cycle progression. The focus cassette consists of three fluorescent fusion proteins (Figure 1A). The foci-forming region (FFR) of Trp53bp1 (53BP1FFR, aa1216–1708) binds to YFP, which is recruited near DSB sites upon DNA damage induction. YFP appears as dot-like clusters (foci), representing a part of the DSB repair machinery (Figure 1B). Similarly, the cell-cycle phase is determined by expressing two different fluorescent proteins, RFP and BFP, fused to hCdt1 (aa 30–120) and hGmnn (aa 1–100), respectively, using the Fucci technique^15^ (Figure 1B).

In this study, a mouse fibroblast cell line (NIH3T3), in which the Focicle cassette was site-specifically knocked in the ROSA26 region using CRISPR-Cas9, was transfected with another vector expressing AsiSI, a type II restriction enzyme isolated from *Arthrobacter* sp. that induces sequence-specific DSBs in the genome. AsiSI cuts the GCGAT/CGC palindromic sequences of mammalian genomic DNA, excluding the CpG methylation sites, causing DSBs. The *AsiSI* gene was fused with an estrogen receptor (ER) to control the timing of activation^16^ by adding 4-hydroxytamoxifen (4OHT), a metabolite of tamoxifen with a high affinity for ER molecules, to the culture medium to control the induction of DSBs by regulating the nuclear translocation of AsiSI (Figures 1C and 1D). As previously reported,^17^ cells had a slight baseline foci level without 4OHT (Figure 1E); the addition of 4OHT showed a dose-dependent increase, best fitted with a simple linear regression (y = 0.06551 x + 0.9514; $r^{2}$ = 0.9934) (Figure 1E). Dose-dependent growth arrest was correlated with DSB levels (Figure 1F). Cell-cycle arrest was also confirmed by increased accumulation of hCdt1 and hGmnn at higher 4OHT concentrations (Figure 1G). This result suggests that the number of DSBs determines the potential for cell proliferation. In this study, we refer to the cumulative load of DSBs during cell growth as the "DSB burden" for clarity.

### Fibroblast monocultures can be explained by logistic growth dynamics

**Figure 2:**
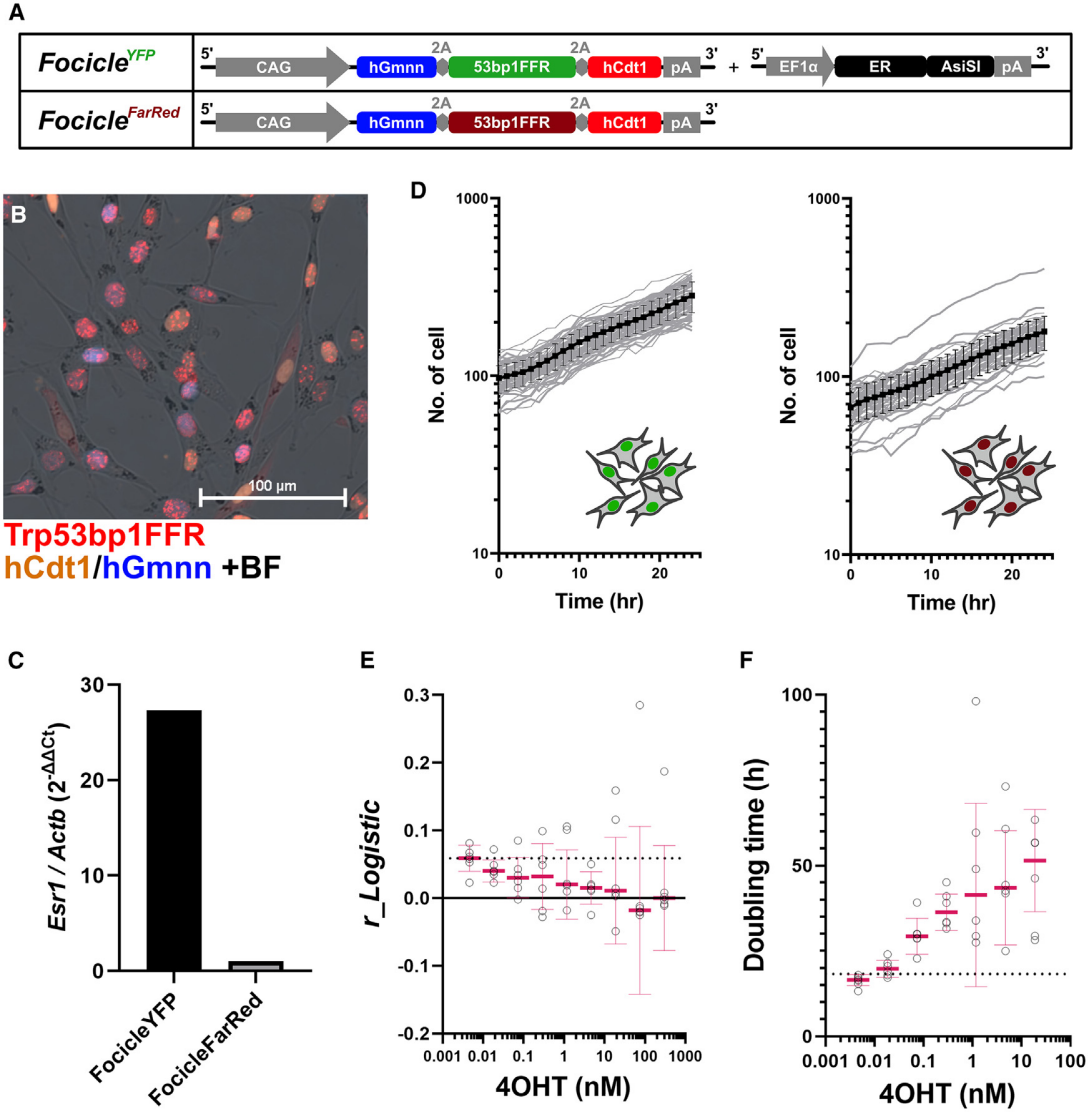
Designs of mixed culture experiments with different genomic stress conditions (A) A new gene cassette with Trp53bp1FFR fused to the fluorescent FarRed (iRFP670) protein was named *Focicle*^*FarRed*^ to distinguish it from a conventional Focicle cassette (*Focicle*^*YFP*^). Both hCdt1/RFP and hGmnn/BFP have the same sequences. (B) The merged image of fluorescence and bright-field (BF) images of NIH3T3 cells expressing the Focicle^FarRed^ cassette captured by live-cell imaging 1 h after 1 Gy of X-ray irradiation. Scale bars, 100 μm. (C) The expression of the estrogen receptor 1 (*Esr1*) was quantified by qRT-PCR and normalized using a housekeeping gene (*Actb*). Gene expression levels were compared with those of Focicle^FarRed^ (2^−^^Δ^^Δ^^Ct^). (D) Focicle^YFP^ (left) and Focicle^FarRed^ cells (right) were monoculture, and time-lapse live-cell imaging was performed every 1 h for 24 h. Gray lines indicate the growth of each 60 replicates sample, and solid black lines and error bars indicate their mean ± SD. (E) Intrinsic natural growth rate for logistic growth estimated from the growth data of Focicle^YFP^ cells after treatment with different concentrations of tamoxifen (4OHT). White circles indicate six replicates points, and solid magenta line and error bars indicate median ± SD. Dotted line indicates the median in the absence of 4OHT treatment. (F) Doubling time for each 4OHT concentration estimated from the growth data of Focicle^YFP^ cells after treatment with different concentrations of 4OHT. White circles indicate six replicates points, and solid magenta line and error bars indicate median ±S.D. Dotted line indicates the median in the absence of 4OHT treatment.

Next, we examined the methods for detecting changes in cell composition in the presence of clones with different DSB burdens. To distinguish cells with or without DSB burden, we synthesized a 53BP1FFR region connected to the FarRed (iRFP670) fluorescent protein (Figure 2A). We then established two cell lines originating from the same cell line but with different fluorescent proteins: one cell line expressed YFP-53bp1FFR and AsiSI^ER^, and another cell line expressed FarRed-53bp1 without AsiSI^ER^ (Figure 2B). As expected, YFP^+^ cells stably expressed the ER (Esr1) that fused with AsiSI, compared to FarRed^+^ cell line, indicating AsiSI is specifically activated in the YFP^+^ cell line (Figure 2C). These two cell lines were used in subsequent experiments while maintaining passages in a monoculture.

The growth curves of YFP^+^ and FarRed^+^ monocultures (60 replicates) for 24 h in the absence of 4OHT are shown in Figure 2D. The number of cells (YFP^+^ or FarRed^+^ nuclei detected per captured image) monotonically increased from the beginning of the observation period (Figure 2D). We then examined mathematical models to fit these biological plots, assuming exponential or logistic cell growth.

When assuming exponential growth, we applied the following Equation 1:

(Equation 1) $$\frac{dN_{i}\left(t\right)}{dt}=r_{Ei}^{T}N_{i}\left(t\right)\;\left(i=Y,F\right)\text{,}$$

where $N_{i}\left(t\right)$ is the number of cells at observation t, and $r_{Ei}^{T}$ is the growth rate at a certain concentration of 4OHT T. The subscripts Y and F denote YFP^+^ and FarRed^+^ cells, respectively.

If the cells grew according to logistic proliferation, Equation 2 was applied:

(Equation 2) $$\frac{dN_{i}\left(t\right)}{dt}=r_{Li}^{T}N_{i}\left(t\right)-a_{i\leftarrow i}^{T}N_{i}{\left(t\right)}^{2}\;\left(i=Y,F\right)\text{,}$$

where $N_{i}\left(t\right)$ is the number of cells at observation t, $r_{Li}^{T}$ is the intrinsic natural growth rate at a certain concentration of 4OHT T, and $a_{i\leftarrow i}^{T}$ is the intraspecific competition coefficient at a certain concentration of 4OHT T, which means the effect of interaction between cells of the same species. The value of $a_{i\leftarrow i}^{T}$ allows for both positive and negative values. When $a_{i\leftarrow i}^{T}$ is positive, it indicates interactions between cells of the same inhibit growth. Conversely, when $a_{i\leftarrow i}^{T}$ is negative, it suggests that growth is promoted. Statistical analysis was performed using the Akaike information criterion (AIC) to determine the suitable model for the fitting results.

For the YFP^+^ monoculture, 40 of 60 wells were selected for logistic growth (Table S1). Similarly, in the case of FarRed^+^ monoculture, 37 of 60 wells were selected for logistic growth (Table S2). When picking up samples showing exponential growth, the doubling time (DT) calculated from the number of cells from t = 0 to t = 24 h was 15.6 ± 2.0 h for YFP^+^ cells and 17.2 ± 1.9 h for FarRed^+^ cells, indicating a slightly slower growth of FarRed^+^ cells (Tables S1 and S2; Figure 2D).

Next, cell growth following various 4OHT treatments was evaluated. As YFP^+^ cells were 4OHT-sensitive, the growth of YFP^+^ cells was suppressed in a dose-dependent manner after 4OHT treatment, as expected. In the case of YFP^+^ monocultures, the intrinsic natural growth rate for the logistic curve was $r_{LY}^{0}$ = 0.063 ± 0.018 and DT = 17.6 ± 1.6 without 4OHT treatment (median ± SD). The $r_{LY}^{T}$ decreased in a dose-dependent manner after 4OHT treatment, indicating a longer DT (Figures 2E and 2F).

### Mixed culture having different DSB burden behaves along with the Lotka–Volterra competition equation

**Figure 3:**
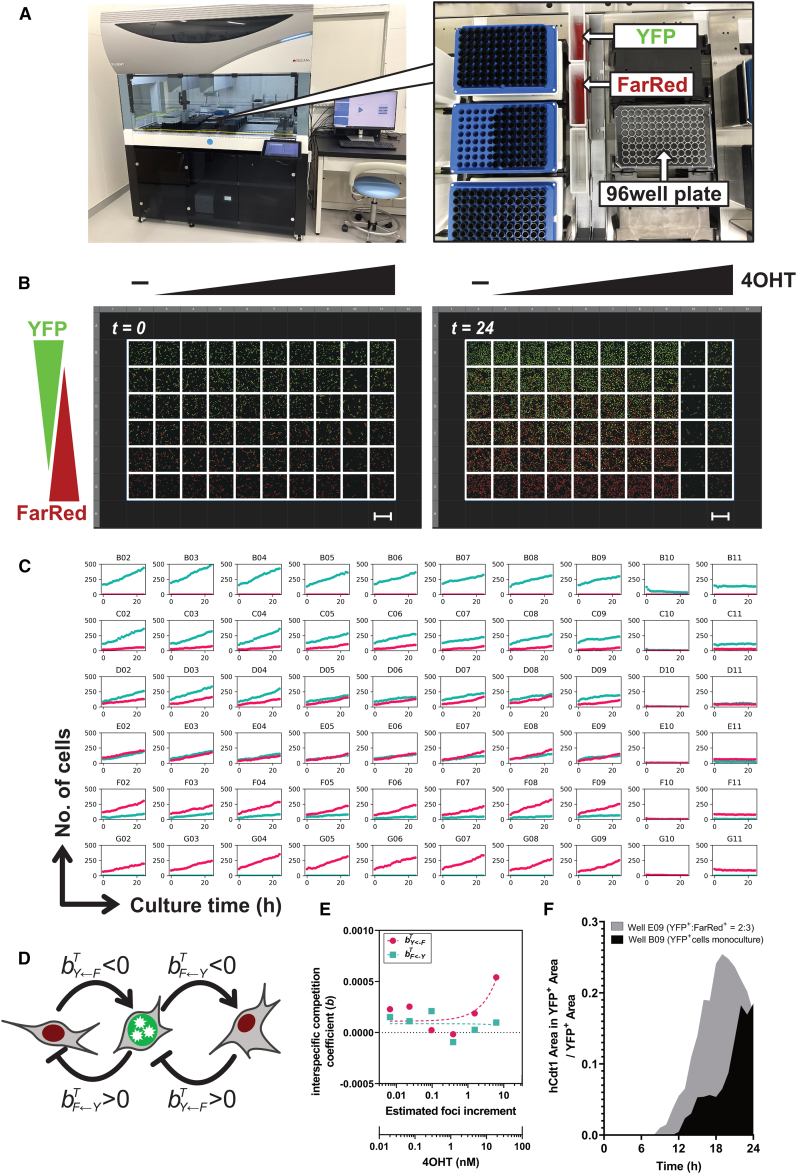
Automated tamoxifen treatment and cell mixing to obtain precise parameters for mathematical models (A) Cell dispensing scheme using the automated liquid-dispensing workstation. Suspensions of YFP^+^ and FarRed^+^ cells were dispensed in varying volumes from troughs into a single 96-well glass-bottom plate. (B) Cells were gradually dispensed into 60 (6 × 10) wells with different mixing ratios: 100% (top row) to 0% (bottom row) for YFP^+^ cells and 100% (bottom row) and 0% (top row) for FarRed^+^ cells. Media containing tamoxifen (4OHT) at serial dilutions (1:4) were added to the plate. Images captured at the start (t = 0 h) and end (t = 24 h) of the time-lapse imaging are displayed as connected tiling images. Scale bars, 500 μm. (C) Time series (0–24 h) of the number of cells in the images of individual wells obtained from the glass-bottom plate shown in (B). Each title denotes the well position. Cyan and magenta dots represent YFP^+^ and FarRed^+^ cells, respectively. (D) Interspecific competition coefficient (b) between FarRed^+^ (red) and YFP^+^ cells (green). The arrow and blunt-ended lines represent the direct activation and inhibition of cell-to-cell interactions, respectively, upon growth stimulation. (E) Interspecific competition coefficients between YFP^+^ and FarRed^+^ cells under various 4OHT treatments, as speculated by intraspecific competition coefficients under monoculture conditions. (F) Time course of fluorescent intensity of hCdt1(RFP^+^) per nuclear area in YFP^+^ cells in the E09 well (gray) or B09 well (Black).

Next, we performed experiments in which the DSB burden was imposed only on the YFP^+^ cells in a mixed culture. Here, we designed a unique experimental setup in which various levels of genomic stress were analyzed in a single assay. First, the distribution of genomic stress in each cell was determined using serial dilutions of 4OHT. We adjusted the mixing ratio of YFP^+^ and FarRed^+^ cells to varying cell components with genomic stress in the cell population. To accurately seed the cells and treat them with 4OHT in a 96-well plate, we utilized an automated liquid-dispensing workstation (Figure 3A). To confirm the dispensing accuracy, we uniformly dispensed YFP^+^ or FarRed^+^ cells into 60 wells in a 96-well glass-bottom plate, where there was an optimal thermal distribution (Figure S1A). Next, images were taken from a centered 60 well using a confocal microscope (Figure S1B), which confirmed that the number of cells showed good accuracy (Figure S1C). Next, YFP^+^ and FarRed^+^ cells were dispensed, except in the periphery well (36 wells) of the 96-well glass-bottom plate, so that the mixing ratio of YFP^+^:FarRed^+^ cells was 10:0, 8:2, 6:4, 4:6, 2:8, and 0:10 (Figure 3B). The next day, a medium for 10 serial dilutions (1:4) of 4OHT to a final concentration of 300 nM to 4.6 pM (nine series and no 4OHT wells) was added, and time-lapse live-cell imaging was immediately performed for 24 h, acquiring 25 sequences of images from 60 wells every hour (Figure 3B). Software image analysis was performed on the 1,500 images obtained in each experiment to determine the cell number and other parameters in YFP^+^ and FarRed^+^ cells (Figure 3C).

Next, mathematical fitting was applied to analyze mixed fibroblast cultures with different DSB burdens. Since the growth of YFP^+^ and FarRed^+^ monocultures typically followed a logistic growth pattern, even with low initial cell numbers, we assumed logistic growth in the mixed culture.^18^^,^^19^ The impact of cell-to-cell interactions was estimated by applying the Lotka–Volterra competition equation as follows:

(Equation 3a) $$\frac{dN_{F}\left(t\right)}{dt}=r_{LF}^{T}N_{F}\left(t\right)-a_{F\leftarrow F}^{T}N_{F}{\left(t\right)}^{2}-b_{F\leftarrow Y}^{T}N_{F}\left(t\right)N_{Y}\left(t\right)$$

(Equation 3b) $$\frac{dN_{Y}\left(t\right)}{dt}=r_{LY}^{T}N_{Y}\left(t\right)-a_{Y\leftarrow Y}^{T}N_{Y}{\left(t\right)}^{2}-b_{Y\leftarrow F}^{T}N_{Y}\left(t\right)N_{F}\left(t\right)$$

where $N_{F}\left(t\right)$ and $N_{Y}\left(t\right)$ represent the numbers of FarRed^+^ and YFP^+^ cells at time *t*, respectively. We assumed that each parameter can vary depending on the concentration of 4OHT T. $r_{LF}^{T}$ and $r_{LY}^{T}$ denote the intrinsic natural growth rate of FarRed^+^ and YFP^+^ cells, respectively; $a_{F\leftarrow F}^{T}$ and $a_{Y\leftarrow Y}^{T}$ are the intraspecific competition coefficients of FarRed^+^ and YFP^+^ cells, respectively; $b_{F\leftarrow Y}^{T}$ and $b_{Y\leftarrow F}^{T}$ are the interspecific competition coefficients. $b_{F\leftarrow Y}^{T}$ represents the influence of YFP^+^ cells on FarRed^+^ cells, whereas $b_{Y\leftarrow F}^{T}$ represents the influence of FarRed^+^ cells on YFP^+^ cells. The superscript T of each parameter represents the concentration of 4OHT. If $b_{Y\leftarrow F}^{T}$ is negative (<0), FarRed^+^ cells will stimulate the cell growth of YFP^+^ cells. If $b_{Y\leftarrow F}^{T}$ is positive (>0), FarRed^+^ cells will suppress the cell growth of YFP^+^ cells (Figure 3D). When the $b_{F\leftarrow Y}^{T}$ and $b_{Y\leftarrow F}^{T}$ values of the mixed culture were normalized by the $b_{Y\leftarrow Y}^{T}$ or $b_{F\leftarrow F}^{T}$ obtained by monoculture at the same concentration of the same plate, the effect of YFP^+^ cells on FarRed^+^ cells remained constant depending on the 4OHT concentration, whereas the effect of FarRed^+^ cells on YFP^+^ cells increased, showing growth inhibition at 4OHT concentrations above 1 nM (Figure 3E). The results of the parameter sensitivity analysis of the model suggest that the uncertainty in the measurement accuracy of these parameters could significantly affect the model’s predictions (Data S1 and Figure S2). This suggests that FarRed^+^ cells may exert a competitive effect on DNA-burdened YFP^+^ cells. We hypothesized that this inhibitory effect of FarRed^+^ cells is a response to the frequency-dependent genomic stress that occurs in YFP^+^ cells. Thus, we next focused on the gene expression induced in YFP^+^ cells by genomic stress in mixed cultures. To assess the effect of the mixed culture on the cell cycle, hCdt1 fluorescence (RFP) per nuclear area (YFP^+^ area) was measured in YFP^+^ cells. The results showed earlier and stronger accumulation of hCdt1 in the E09 well, which contained 60% FarRed^+^ cells, than in the YFP^+^ monoculture (B09 well), even after treatment with the same 18.75 nM 4OHT (Figure 3F). This finding suggests the presence of factors that act on G1 arrest other than the genomic stress induced by 4OHT treatment.

### Activation of Acta2 exclusively occurs in cells with a DSB burden under competitive cell-to-cell interactions

**Figure 4:**
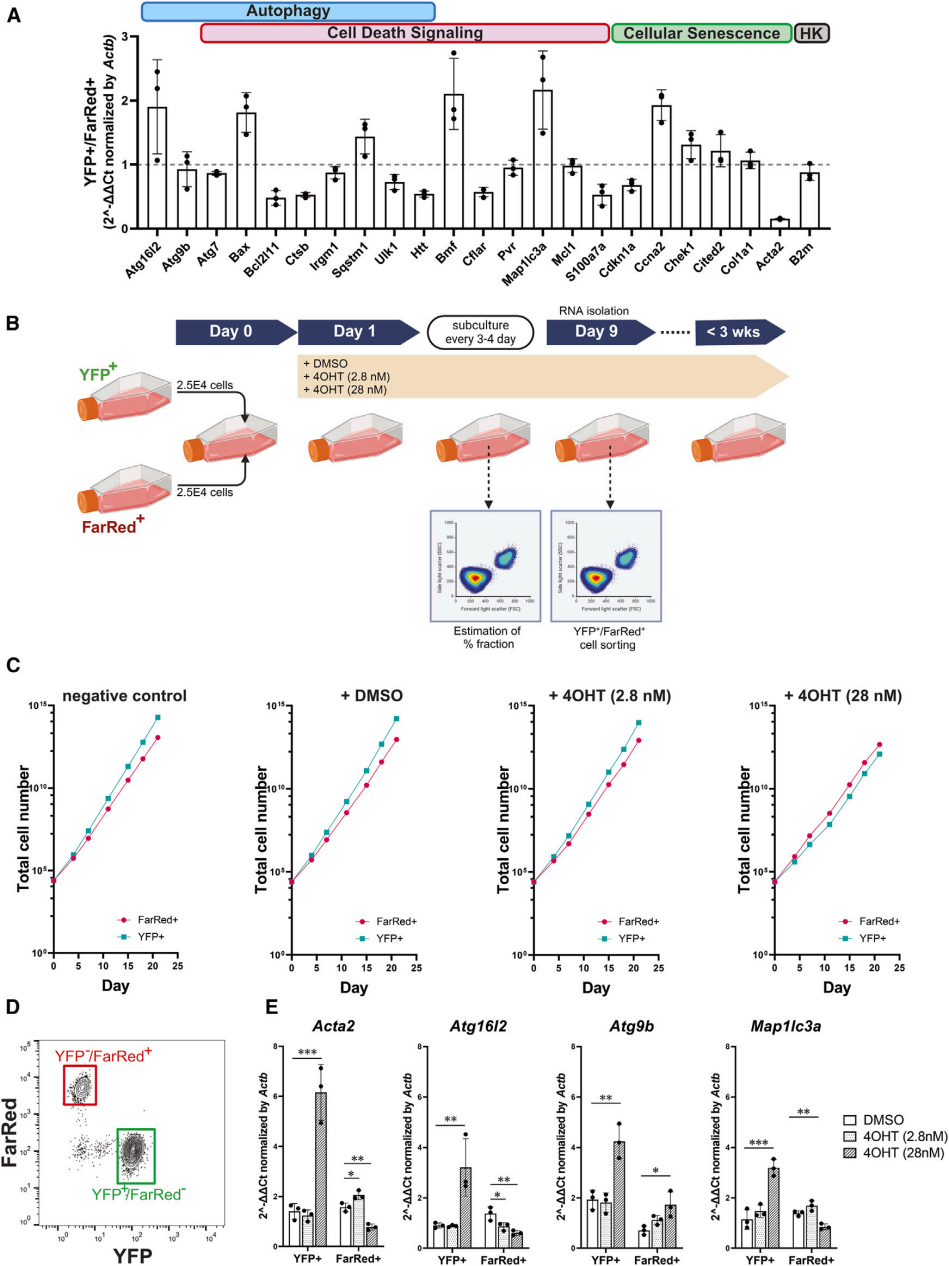
Gene expression under competitive mixed culture containing different genomic stress level (A) Genome stress array using probe-based qRT-PCR. The gene set was chosen from a preliminary screening to correlate autophagy, cell death signaling, and cellular senescence. This array contained two housekeeping (HK) genes (*Actb* and *B2m*) and was normalized to *Actb* expression. The gene expression of YFP^+^ cells compared to FarRed^+^ cells was shown in 2^−^^ΔΔCt^ value as mean ± SD of triplicate in one representative experiment. The dotted line indicates no differential expression (1). (B) Schematic of cell passage and total RNA isolation of 4OHT-treated cells. On day 0, YFP^+^ and FarRed^+^ cells were mixed equally (2.5E4 cells). On Day 1, the media were exchanged for 4OHT (2.8 nM or 28 nM) or DMSO. Medium exchange was used as a negative control. The cells were passaged every 2 or 3 days for approximately 3 weeks. At passage, the fluorescence distributions of the YFP^+^/FarRed^−^ and YFP^−^/FarRed^+^ fractions were measured by flow cytometry, and the remaining cells were subcultured in media containing the same drug concentration. On day 9, total RNA was isolated from the YFP^+^/FarRed^−^ and YFP^−^/FarRed^+^ fractions for qRT-PCR. Illustrations were created with Bioredner.com. (C) Cell growth in the cumulative number of YFP^+^ and FarRed^+^ cells from the start of the mixed culture was calculated from the ratio of YFP^+^ and FarRed^+^ fractions, measured by flow cytometry. (D) Example of sorted gates for the ratio calculation of YFP^+^ and FarRed^+^ cell fractions and total RNA isolation. The image was rebuilt from an FCS file using the FlowJo software. (E) Relative expression of *Acta2*, *Atg16l2*, *Atg9b*, and *Map1lc3a* genes normalized by *Actb* in YFP^+^/FarRed^−^ or YFP^−^/FarRed^+^ fractions were isolated from DMSO, 4OHT (2.8 nM), and 4OHT (28 nM) and expressed as mean ± SD of triplicate in one representative experiment. The 2^–ΔΔCt^ value was calculated using baseline expression of the same fractions isolated from the negative control. ∗*p* < 0.05, ∗∗*p* < 0.01, ∗∗∗*p* < 0.001.

An important cell fate determination of cellular responses to DNA damage is driven by two effector pathways: p53 and pRB^20^ signaling. Autophagy is activated by a p53-mediated transcriptional response.^21^ Cells that escape autophagy or death survive and undergo senescence.^22^ Therefore, we examined the gene set expression of autophagy, cell death, and cellular senescence, which are critical for determining the cellular pathways under genomic stress. To compare the baseline cellular status between cell lines, we first evaluated gene expression in a monoculture of YFP^+^ and FarRed^+^ cells without 4OHT using a polymerase chain reaction (PCR) array (Figure S3). Among the gene sets of cellular senescence activated in YFP^+^ cells in comparison with FarRed^+^ cells, almost all 69 genes were detected within a 3-fold range of expression (Figures S3A and S3B). When genomic stress was applied during mixed culture, a preliminary comparison of the expression of YFP^+^ and FarRed^+^ cells treated with 300 nM 4OHT showed a change in expression in the direction of activation for many autophagy- and cell-death-signaling-related genes in YFP^+^ cells. Based on the preliminary screening, we evaluated 23 representative gene sets associated with stress-induced DNA damage responses related to autophagy, cellular senescence, and cell death pathways using a genomic stress array (Figure 4A). In YFP^+^ cells, only the *Acta2* gene showed lower expression levels in YFP^+^ cells than in FarRed^+^ cells (Figure 4A). Next, the same number of YFP^+^ and FarRed^+^ cells were mixed and subcultured for a long period (within 3 weeks) in the presence of 4OHT (2.8 nM or 28 nM), where a decrease in proliferative capacity and an increase in foci formation were observed, or DMSO as a solvent for 4OHT (Figure 4B). The transition of the total cell number from the beginning of cell culture was calculated based on the ratio of the YFP^+^ and FarRed^+^ fractions. The results showed that cell growth was maintained for 3 weeks, but only 28 nM 4OHT treatment resulted in a reduced ratio of YFP^+^ cells (Figure 4C), equivalent to the results of 24-h time-lapse imaging. The growth of YFP^+^ cells was completely terminated by day 10 in the presence of 300 nM 4OHT (Figure S4). On day 9, the YFP^+^ and FarRed^+^ fractions were collected by cell sorting (Figure 4D), and gene expression was normalized to the negative control obtained from the same gates. In YFP^+^ and FarRed^+^ cells, neither 2.8 nM nor 28 nM 4OHT treatment showed drastic differences compared to the DMSO-treated control (Figure S5). In contrast, at 28 nM 4OHT, the expression of *Atg16l2, Atg9b, Map1lc3a* and *Acta2* increased (>3-fold) only in YFP^+^ cells; in particular, the increase in *Acta2* expression was significant (Figure 4E).

## Discussion

This study devised a data-driven approach using mathematical models for decision-making on prediction of cell-to-cell interactions under specific genomic stresses. The results indicated that a particular genomic stress would activate fibroblasts, transforming them into CAFs. Recent research has shown that the activation of fibroblasts within the ECM plays a notable role in the development of the cancer microenvironment, as well as tumor invasion and metastasis.^23^^,^^24^^,^^25^ Therefore, CAFs are of particular importance in cancer therapy and prevention. CAFs are heterogeneous, with various subsets within tissues,^26^ which may contribute to the difficulty in understanding the cancer microenvironment. Understanding the molecular functions of CAFs will provide important information for cancer therapy.

*Acta2* expression in fibroblasts has been linked to various cellular processes, including differentiation and expression of myofibroblast markers. Another study on fibroblast heterogeneity and differentiation models in breast tissue identified *Acta2* as a marker gene associated with fibroblast activation and mesenchymal cell differentiation.^27^ In addition, single-cell RNA sequencing data showed that Acta2 is a myofibroblast marker associated with tumor progression and fibrosis.^28^ Besides enzymatic digestion, a study of radiation-induced early differentiation of human fibroblasts showed that the expression of Acta2 was upregulated with delayed kinetics on days 5–6, which demonstrated a correlation between the *Acta2* gene and cell growth.^29^

Interestingly, genes related to autophagy (*Atg9b, Atg16l2, Map1lc3a*), which are associated with inflammation and immune responses in the skin, were activated only in 28 nM YFP^+^ cells. This finding suggests that genomic stress-inducible autophagy and inflammation are correlated with CAF development.

Studies have been conducted to understand competitive cell-to-cell interactions as mathematical models indexed by cell growth through evolutionary game theory. Kaznatcheev et al. reported that a mixed culture of cancer cells, and CAFs in the exponential growth phase was interpreted from replicator dynamics. It was found that the presence of CAFs modifies the effect of therapeutic drug treatments.^30^ Farrokhian et al. devised a predictive model of the therapeutic effect of the competitive evolution of resistant strains owing to different frequency-dependent interactions of drug resistance.^31^

The treatment with 2.8 nM or 28 nM 4OHT induced approximately 0.19 and 1.9 increments in 53bp1 large foci, respectively (Figure 1F). Consecutive DSB induction by 2.8 nM and 28 nM 4OHT affected cell growth; however, only 28 nM treatment increased *Acta2* expression. From these results, we considered the possibility that the consecutive induction of 1.9 large foci on average may direct normal fibroblasts toward aberrant properties, such as CAFs. We previously reported that the large foci detected using our evaluation system (approximately 8 foci per Gy) corresponded to approximately 20% of the theoretical DSBs.^17^ Thus, although a rough estimate, treatment with 28 nM 4OHT is projected to yield approximately 10.3 DSBs per cell. Prior studies indicated that exposure to 300 nM 4OHT resulted in 80 DNA cuts,^32^ confirming our estimation of 98 foci at 300 nM. As AsiSI digestion induces DSBs constitutively, precise quantification of DSBs per unit of time presents challenges. Nevertheless, if these estimates hold true in different scenarios, we can compare the impact of various DSB-inducing agents on cancer microenvironment formation. For example, the DSB yields of various radiations and chemicals have been reported.^33^^,^^34^ In the future, understanding the quantitative relationship between environmental factors and cancer promotion risk using experimental models that regulate DNA-damaging agents will allow us to assess their correlation with the cancer-associated microenvironment.

### Limitations of the study

In this study, we established a streamlined process to identify interactions and activating molecules based on the correlation between cell count and DNA damage in fibroblasts. Given that we used clones derived from a mouse fibroblast cell line, it will be necessary to verify whether similar interactions occur in other clones or in human fibroblasts in the future studies. Additionally, direct evidence is still lacking that DNA-damage-induced CAF formation enhances the malignancy of cancer cells within actual microenvironments. Further validation using a co-culture system of fibroblasts and cancer cells will be essential to develop a more comprehensive mathematical model of cancer progression at the tissue level.

## Resource availability

### Lead contact

Further information and requests for resources and reagents should be directed to and will be fulfilled by the lead contact, Kensuke Otsuka (ohken@criepi.denken.or.jp).

### Materials availability

All the cell lines and plasmids used in this manuscript will be made available from the lead contact upon request.

### Data and code availability

• Data: microscopy data reported in this paper will be shared by the lead contact upon request.
• Code: Mathematical models and fitting datasets were deposited on Mendeley at https://doi.org/10.17632/n2xn3wvw2w.1
• Additional information: any additional information required to reanalyze the data reported in this paper is available from the lead contact upon request.

## Acknowledgments

We thank Chiharu Furukawa for plasmid construction. We also thank Tomoko Ueno of 10.13039/501100001697Keio University for supporting cell sorting. We thank all the members of the Biology and Environmental Chemistry Division of CRIEPI for their support. This work was partially supported by Grants-in-Aid for Scientific Research (19H04274 and 20K19972) from the Japan Society for the Promotion of Science (JSPS) and the Network-type Joint Usage/Research Center for Radiation Disaster Medical Science. We would like to thank Editage (www.editage.jp) for English language editing.

## Author contributions

K.O. designed the study, including the construction of the plasmids, methods for live-cell imaging and analysis, and scripts for an automated liquid-handling workstation. K.U. designed the mathematical models and performed data fitting. Y.Y. performed cell culture and plate formatting using an automated liquid-handling workstation and quantitative RT-PCR and processed data from flow cytometry and image acquisition after live-cell imaging. K.O., K.U., and Y.Y. performed the data analysis. A.S. designed the genomic stress model using the AsiSI^ER^ system. K.O., K.U., Y.Y., and A.S. prepared the manuscript.

## Declaration of interests

The authors declare no competing interests.

## Declaration of generative AI and AI-assisted technologies

During the preparation of this manuscript, the authors utilized ChatGPT and DeepL to improve the readability and language of the manuscript. Following the use of these tools, the entire manuscript was thoroughly reviewed and edited by the authors, with additional English language editing performed by Editage. The authors take full responsibility for the content of this publication.

## STAR★Methods

### Key resources table

**Table:** 

REAGENT or RESOURCE	SOURCE	IDENTIFIER
**Chemicals, peptides, and recombinant proteins**		

4-hydroxytamoxifen	Sigma-Aldrich	Cat #H7904

**Critical commercial assays**		

GeneArt Seamless Cloning and Assembly Kit	Thermo Fisher Scientific	Cat #A13288
SG cell line solution	Lonza	Cat #PBC3-00675
SimplyRNA Kit	Promega	Cat #AS1390
RT^2^ profiler PCR arrays	QIAGEN	Cat #330231
Mouse Autophagy panel	QIAGEN	Cat #PAMM-084ZA
Mouse Cell Death Pathway Finder panel	QIAGEN	Cat #PAMM-212ZA
Mouse Cellular Senescence panel	QIAGEN	Cat #PAMM-050ZA
RT^2^ first strand Kit	QIAGEN	Cat #330404
PrimeScriptII 1^st^ strand cDNA Synthesis Kit	Takara Bio Inc.	Cat #6210
PrimeTime qPCR probe Assays	Integrated DNA Technologies	N/A

**Deposited data**		

*Focicle*^*YFP*^	Otsuka et al.^17^	N/A
*Focicle*^*FarRed*^	This study	N/A
pPB-EF1a-ER/AsiSI-Puro^R^	This study	N/A
Python scripts and datasets	This study	https://doi.org/10.17632/n2xn3wvw2w.1

**Experimental models: Cell lines**		

NIH3T3	ATCC	Cat #CRL-1658; RRID: CVCL_0594

**Software and algorithms**		

GraphPad PRISM (version 8.4.3)	GraphPad Software	https://www.graphpad.com
FlowJo (version 10.10.0)	BD	https://www.flowjo.com
Morpheus	Broad Institute	https://software.broadinstitute.org/morpheus/
Jupyter notebook	Jupyter	https://jupyter.org
FluentControl (version 3.3)	Tecan	N/A
QuantStudio Design & Analysis (version 1.5.3)	Thermo Fisher Scientific	N/A

### Experimental model and study participant details

#### Cell line

Mouse NIH3T3 cells (#CRL-1658, RRID: CVCL_0594, male) were purchased from ATCC (Manassas, VA, USA). Cells were subcultured two times every week in Dulbecco’s Modified Eagle’s Medium/Nutrient Mixture F-12 (#D8437, Sigma-Aldrich, St. Louis, MO, USA) with 10% Bovine Serum (#16170-078, Thermo Fisher Scientific, Waltham, MA, USA) and 1X Penicillin–Streptomycin (#154140-122, Thermo Fisher Scientific) in a T75 flask (#430641U, Corning Inc., Corning, NY, USA). At passage, the cells were washed once with 10 mL of Dulbecco’s phosphate-buffered saline (PBS(−)) (#08192, Shimadzu Diagnostics Corporation, Tokyo, Japan), washed with 1 mL of 0.05% trypsin-EDTA (#25300-054, Thermo Fisher Scientific), and allowed to detach in a CO_2_ incubator. Then, 9 mL of complete medium was added, pipetted, and transferred to 15 mL conical tubes (#188271, Greiner bio-one, Kremsmünster, Austria). An equal volume of the cell suspension and 0.4% Trypan Blue stain (10 μL each) was mixed well, and cell density was determined on a Countess II cell counter (Thermo Fisher Scientific). Cells were resuspended in 15 mL of prewarmed complete medium in a new T75 flask at 1–5 × 10^4^ cells per passage interval, mixed well to spread cells uniformly, and incubated at 37°C with 5% CO_2_.

### Method details

#### Plasmid construction

Construction of the *Focicle*^*YFP*^ cassette has been previously reported.^17^ Briefly, the coding sequences for the foci-forming region (FFR, aa1216-1708) of *Trp53bp1*, *hCdt1* (aa30-120), and *hGmnn* (aa1-100) were fused to YPet (YFP), mRuby3 (RFP), and mTagBFP2 (BFP), respectively, and combined with 2A peptides to express tricistronically under the CAG promoter (Figure 1A). The three components were ligated using Seamless Cloning (Thermo Fisher Scientific) into the pUC19 backbone vector flanked by the 5′ and 3′ arms of mouse ROSA26 region. The constructed plasmid was then transformed into *E. coli* (HST08). Similarly, we designed a new Trp53bp1 FFR fused with iRFP670 (FarRed) instead of YFP to construct a *Focicle*^*FarRed*^ cassette (Figure 2A).

#### Transfection and cloning

Cotransfection with plasmid vectors was performed via electroporation using a 4D-NucleoFector system (Lonza, Basel, Switzerland) to establish fluorescent cell lines. Passaged cells were collected in 15 mL conical tubes and centrifuged at 100 × *g* for 5 min to obtain a pellet of 10^6^ cells. The cell pellet was resuspended with 100 μL of SG cell line solution (#PBC3-00675, Lonza) containing *Focicle*^*YFP*^ or *Focicle*^*FarRed*^ vector, as well as sgRNA/Cas9 vector, targeting the ROSA26 region. The mixed suspension was transferred to a single cuvette. Electroporation was performed under the #EN158 pulse code and resuspended in a complete medium. The next day, YFP^+^ or FarRed^+^ fluorescent-expressing cells were isolated using the MoFlo AstriusEQ Cell Sorter System (Beckman Coulter, Brea, CA, USA).

For 4OHT-dependent DSB generation, a piggyBac transposon vector expressing *AsiSI*^*ER*^ (pPB-EF1a-ER/AsiSI-Puro^R^) was constructed and co-transfected with Super PiggyBac transposase (#PB210PA-1, System Biosciences, Palo Alto, CA, USA), as described above. Single-cell sorting after selection with 2 μg/mL puromycin (#A1113803, Thermo Fisher Scientific) was performed to obtain clones expressing both YFP and 4OHT responsiveness.

#### Automated liquid-handling

For accurate dispensing of cells in the culture mixture and 4OHT addition, we used an automated liquid-handling system, Fluent780 (Tecan, Männedorf, Switzerland). YFP^+^ and FarRed^+^ cells were detached from the flask, and cell density was estimated as described above. Each cell line was collected in 50 mL centrifuge tubes (#430829, Corning) and transferred to 100 mL troughs by dilution with complete medium to adjust the concentration to 2,500 or 10,000 cells/well on a 96-well glass-bottom plate after dispensing (Figure 3A). To fill the unused periphery (36 wells) of the 96 well plate, 15 mL PBS(−) was added to another 100 mL trough (Figure 3A). For monocultures, the two troughs contained the same cells (YFP^+^/YFP^+^ or FarRed^+^/FarRed^+^), while for mixed cultures, the two troughs contained different cells (YFP^+^/FarRed^+^) (Figure 3A). For liquid-handling, two customized scripts were created using Fluent Control (Ver. 3.3, Tecan), namely, the cell dispensing operation on day 0 and 4OHT addition on the next day (day 1 or day 2), as follows.

On day 0, the cells were seeded in a 96-well SensoPlate (#655892, Greiner Bio-One) under the cell dispensing operation. First, considering the optimal temperature distribution of the microscope stage chamber, we dispensed 160 μL of PBS(−) to the peripheral 36 wells out of the 96 wells and excluded them from observation. Next, we seeded 160 μL of YFP^+^ suspension to Column B, 128 μL to Column C, 96 μL to Column D, 64 μL to Column E, 32 μL to Column F, and 0 μL to Column G to change the mixing ratio of cells per well. Vice versa, we seeded 0 μL of FarRed^+^ cells to B, 32 μL to C, 64 μL to D, 96 μL to E, 128 μL to E, and 160 μL to G, giving rise to 160 μL in every well. This created six patterns of mixing ratio by YFP^+^ and FarRed^+^ cells: 1:0, 4:1, 3:2, 2:3, 1:4, and 0:1. After the cell dispensing operation, the cells were allowed to stand at room temperature (approximately 24°C) for 30 min and then incubated overnight at 37°C and 5% CO_2_ (cultured cell plate).

On day 1, a plate was prepared for serial dilutions of the drug. First, 4-hydroxytamoxifen (4OHT) was dissolved in dimethyl sulfoxide (DMSO) (#D8418, Sigma-Aldrich) as a solvent to prepare a 37.5 mM stock solution and stored in a 2 mL tube (#1394-200-MS, Watson Co., Ltd., Kobe, Japan). One empty 2 mL tube for dilution, an empty 96-well Hard-Shell microplate (#HSP9611, BioRad Laboratories Inc., Hercules, CA, USA) for serial dilution, and a trough (#30055743, Tecan) containing 25 mL of complete medium was set in the designated position in Fluent780. A script was then run to dispense 90 μL/well of medium from the trough into 60 wells of B2-G11. Meanwhile, in an empty 2 mL tube, 420 μL of medium and 80 μL of 37.5 mM 4OHT were dispensed and agitated ten times to 7.5 mM; then, 30 μL/well was dispensed into B11-G11 wells, followed by pipetting ten times to make a well-mixed 4-fold dilution. Next, 30 μL/well each from B11-G11 was aspirated and dispensed to B10-G10 wells and mixed well by pipetting ten times. This operation was repeated up to B3-G3 wells to prepare a 10-step 1:4 serial dilution (drug plate).

Next, the cultured cell plate was moved from the CO_2_ incubator to the deck of the Fluent 780. To prevent cell detachment, 40 μL from each 96-well drug plate was gently added to 160 μL of medium in the same well IDs of the cultured cell plate. Immediately after dispensing, time-lapse live-cell imaging was performed using a confocal laser microscope.

#### High-content time-lapse live-cell imaging

High-content time-lapse live-cell imaging was performed using a confocal laser microscope (A1R HD25; Nikon, Tokyo, Japan) equipped with a stage-top incubator (STX; Tokai Hit. Co., Ltd., Shizuoka, Japan). On the day of imaging, the confocal laser microscope, STXG controller, and workstation were turned on to warm for at least 1 h before the measurements. Next, 25 mL of deionized water was placed in the TIZWX chamber pool, and the controller was set to 37°C, 5% CO_2_. Before measurement, a 96-well glass-bottom plate was placed in the stage chamber. Image acquisition and analysis were performed using NIS Elements AR software (ver. 5.2100, Nikon) in confocal mode. To suppress z axis drift during long-term image capture, the Perfect Focus System (PFS) was set to ON, the light path was switched to the confocal unit, and the z-position was adjusted again to confirm the best contrast on the LIVE screen.

Next, settings for a series of time-lapse live-cell imaging were made in the JOBS add-in as follows: “Capture” was set to “Single Image,” z stack was not selected, “Autofocus” was disabled, and “PFS Only” was selected for refocusing. In the “Sample Definition,” “Well Ordering” was set to “Left to Right,” applied to 60 wells from B2 to G11. The objective lens was plan Apoλ10X, and the imaging range was set to the center of the well at a single point (Figure S1A). The time sequence was set to “25” at “Number of Loops,” and “Interval Run” was set to “Every 1 h” “DATA Analysis” was performed “at the same time” at image acquisition by loading a recipe built in the “General Analysis” add-in, which included nucleus recognition, automatic detection of 53bp1 focus, and fluorescence intensity of the cell cycle. After completion of time-lapse imaging and data acquisition, the parameters analyzed were displayed as output in a semicolon-delimited CSV format.

#### Fluorescence-activated cell sorting

Scatter plots were obtained from cells at passage using a MoFlo Astrius EQ Cell Sorter (Beckman Coulter). The ratio of YFP^+^ and FarRed^+^ cells in mixed cultures was calculated as follows: The cell sorter with a 70 μm nozzle and the system status was checked every time using Ultra Rainbow Fluorescent Particles (#B27526, Beckman Coulter). Cell suspensions (1 mL) were filtered through a 35 μm cell strainer tube (#352235, Corning) and set in the cell sorter. We set gates to select the cell population for analysis using X: Forward Scatter (FSC) and Y: Side Scatter (SSC). Next, the scattergram was plotted using X: FSC-Area and Y: FSC-Height signals and gated to a single-cell population for doublet cell removal. The same applies to SSC. The YPet protein was excited by a 488 nm laser, and emission was detected using a 513/26 nm bandpass filter. The iRFP670 protein was excited by a 640 nm laser, and emission was detected using a 671/30 nm bandpass filter. Then, we set single positive gates for YFP^+^/FarRed^−^ and YFP^−^/FarRed^+^. The stream pressure was set in the 1,000–3,000 events/s range, and the gate limit was set to approximately 50,000 events of the main population.

The following methods were used to isolate total RNA from YFP^+^/FarRed^−^ and YFP^−^/FarRed^+^ cells. Before cell sorting, a chiller was turned on, and cell samples were maintained at 4°C. After passing the system check as described above, deflection plates were attached to the cell sorter, and a 1.5 mL Eppendorf tube was set on the droplet receiver. After adjusting the sorting position and charge phase to ensure that the drops fell precisely in the center of the 1.5 mL Eppendorf tube, the automated drop monitoring Intellisort was stabilized. Sorting parameters were as follows: “Abort Mode” was set to “Purify,” “Drop Envelop” was set to “1–2” drops, and collection of YFP^+^/FarRed^−^ cells to “Left1” position and YFP^−^/FarRed^+^ cells to “Right1” position. Immediately before cell sorting, 1.5 mL Eppendorf tubes with ice-cold 500 μL of PBS(−) were set in “Left1” and “Right1” positions. Cells were collected until the sample mixture was empty.

#### Irradiation

The cells were exposed to X-rays using an MBR-1520R4 X-ray generator (Hitachi Power Solutions Co. Ltd., Ibaraki, Japan) operated at 150 kV and 20 mA with a 0.5 mm Al + 0.3 mm Cu filter. The dose-rate was 0.6 Gy/min.

#### Total RNA isolation

Total RNA was extracted from cells using the SimplyRNA Kit (#AS1390; Promega, Madison, WI, USA). The YFP^+^/FarRed^−^ and YFP^−^/FarRed^+^ cells collected from the culture flask or cell sorting were centrifuged at 100 × *g* for 5 min. The pellet was resuspended in a prechilled mixture of 200 μL of Homogenization Solution and 4 μL of 1-Thioglycerol. Then, 200 μL of Lysis Buffer was added to the cell suspensions. The lysed cells were transferred to well #1 of the SimplyRNA Maxwell RSC cartridges. Next, 5 μL of DNase I Solution was added to well #4. DNase/RNase-free water (50 μL) was added to the Elution Tubes. Cartridges and Elution Tubes were placed in a Maxwell RSC instrument (#AS4500). After isolation, the purity and yield of total RNA were checked using a spectrophotometer DS-11 (Denovix, Wilmington, DE, USA) and stored at −80°C until use.

#### PCR array

Three gene panels of a commercially available RT^2^ profiler PCR array (QIAGEN, Venlo, The Netherlands) were used to screen the gene expression levels. To evaluate autophagy, cell death, and cellular senescence, we used panels for Mouse Autophagy (#PAMM-084ZA), Cell Death Pathway Finder (#PAMM-212ZA), and Cellular Senescence (#PAMM-050ZA), respectively. We prepared the master mix (MM) and cDNA (D) plates independently as follows: First, 100 μL of 2X RT^2^ SYBR Green ROX MasterMix was dispensed and vortexed well in an MS3 vortexer (IKA-Works, Inc., Wilmington, NC, USA) to completely dissolve the primers and SYBR Green, which was designated as the MM-plate. Reverse transcription to cDNA was performed using the RT^2^ first strand Kit (#330404, QIAGEN) in a reaction of 15 min at 42°C, followed by 5 min at 95°C. The obtained sample was diluted with RNase-free water, and 35 μL was dispensed into each well of the 96-well PCR plate (#437-177C; Watson Co., Ltd.), designated as the D-plate. Next, we aspirated 5 μL of the contents of the MM-plate and D-plate and mixed them in a new 96-well plate using a table-top dispenser EDR-384SII (Biotec Co. Ltd., Tokyo, Japan) to obtain 10 μL of the reaction solution. The samples were run on a QuantStudio3 real-time PCR system (Thermo Fisher Scientific). The obtained Ct values were entered into a pre-designed Microsoft Excel worksheet (QIAGEN). Genes with low expression levels with Ct values >35 were excluded, and other |FC| ≥ 2 genes were extracted. Tamoxifen-sensitive YFP^+^ cells were used as the test sample, and tamoxifen-resistant FarRed^+^ cells were used as the control sample for calculating gene expression (log2FC) between the two groups.

#### Probe-based qRT-PCR

Total RNA was extracted as previously described. Reverse transcription of cDNA was performed using a PrimeScript II 1^st^ strand cDNA Synthesis Kit (#6210; Takara Bio Inc., Shiga, Japan) in the presence of random 6-mer primers. The concentration of cDNA was approximately 0.2–2 ng/μL (total RNA equivalent). cDNA was mixed with pre-designed primers and probes for PrimeTime qPCR Probe Assays (Integrated DNA Technologies, Inc., Coralville, IA, USA) and 2X MasterMix containing 1/500 ROX reference dye. Finally, 10 μL of the reaction solution was run on a QuantStudio3 real-time PCR system (Thermo Fisher Scientific). Data were analyzed using QuantStudio Design and Analysis Software (ver. 1.5.3), and gene expression of YFP^+^/FarRed^−^ and YFP^−^/FarRed^+^ cells was normalized by housekeeping genes (*Actb* or *B2m*). From the obtained Ct values, the ΔΔCt method was used to compare the differentiation of gene expression to the negative control (neither DMSO nor 4OHT treatment) sample.

#### Mathematical modeling

The Scipy.integrate.odeint module by Python (https://docs.scipy.org/doc/scipy/reference/generated/scipy.integrate.odeint.html) was used to solve ordinary differential equations (Equations 1 and 2), followed by the Scipy optimize curve_fit module by Python (http://docs.scipy.org/doc/scipy/reference/generated/scipy.optimize.curve_fit.html) to fit the number of cells $N_{i}\left(0\right)$, growth rate $r_{Ei}^{T}$ at the beginning of culture for exponential growth, number of cells at the beginning of logistic growth $N_{i}\left(0\right)$, intrinsic natural growth rate $r_{L_{i}}^{T}$, and intraspecific competition coefficient $a_{i\leftarrow i}^{T}$. The initial values for fitting were $r_{Ei}^{T}=0.5$ for exponential growth, $r_{L_{i}}^{T}=1.0$ for logistic growth, and $a_{i\leftarrow i}^{T}=0.001$. For fitting YFP^+^ monoculture, we set $a_{i\leftarrow i}^{T}=0.0$ because the fitting did not converge at 4OHT concentrations of 75 and 300 nM. In all the other cases, $N_{i}\left(0\right)$ was set to the minimum count among the time series in the same well. In the model selection for exponential or logistic growth, AICs were applied to choose a suitable model for eliminating common terms by assuming a normal distribution as the error distribution.

(Equation 4) $$AIC=S\;\log\left(\frac{1}{S}\sum_{t}{\left(N_{i,t}-N_{i}\left(t\right)\right)}^{2}\right)+2k$$

where S is the number of observation time points for each well; $N_{i,t}$ is the number of cells at the time of observation; $N_{i}\left(t\right)$ is the value estimated by the model at the time corresponding to the observation; k is the number of parameters (k=1 for exponential growth and k=2 for logistic growth). The model with a smaller AIC between the compared models was considered the better fitting.

Cell-to-cell interactions between YFP^+^ and FarRed^+^ cells in mixed culture were estimated using the Lotka–Volterra competition equations (Equations 3a and 3b). In the equations, we applied intrinsic natural growth rate $r_{L_{i}}^{T}$ and intraspecific competition $a_{i\leftarrow i}^{T}$ from the value estimated by the monoculture (i.e., B2-B11 for YFP^+^ cells and G2-G11 for FarRed^+^ cells) under the same dose of 4OHT treatment. The Scipy.integrate.odeint module by Python (https://docs.scipy.org/doc/scipy/reference/generated/scipy.integrate.odeint.html) was used to solve ordinary differential equations (Equations 3a and 3b), followed by fitting with the number of cells at the beginning of culture $N_{i}\left(0\right)$ and intraspecific competition coefficient $b_{Y\leftarrow F}^{T}$, $b_{F\leftarrow Y}^{T}$ using the Scipy.optimize.curve_fit module by Python (http://docs.scipy.org/doc/scipy/reference/generated/scipy.optimize.curve_fit.html). The initial value of $N_{i}\left(0\right){,b}_{Y\leftarrow F}^{T}$ and $b_{F\leftarrow Y}^{T}$ was applied to the initial value of the number of cells at t=0, $a_{Y\leftarrow Y,}^{T}$ and $a_{F\leftarrow F}^{T}$, respectively.

### Quantification and statistical analysis

To generate line, dot, and bar graphs, we utilized PRISM software (version 8.4.3, GraphPad Software, LLC, La Jolla, CA, USA). Data visualization, including heatmaps and scatter plots, was performed using Morpheus (https://software.broadinstitute.org/morpheus), FlowJo (version 10.10.0), or Python programs within the Jupyter Notebook environment (https://jupyter.org). The analysis of RT^2^ Profiler PCR arrays involved gene expression calculations conducted with a Microsoft Excel worksheet provided by the manufacturer. To determine significant differences (α = 0.05) in gene expression between the test groups (2.8 or 28 nM 4OHT) and the control group (DMSO), we employed one-way ANOVA (Dunnett’s multiple comparisons test) using PRISM software.
