# Supplementary material for: Prediction of key biological processes from intercellular DNA damage differences through model-based fitting
Source: iScience. 2024 Nov 26;27(12):111473. doi: 10.1016/j.isci.2024.111473 (PMC11667071; doi:10.1016/j.isci.2024.111473)
Supplement: Document S1. Figures S1–S5 and Tables S1 and S2 and Data S1 [file mmc1.pdf]

iScience, Volume *27*

Supplemental information

Prediction of key biological processes from intercellular DNA damage differences through model-based fitting

Kensuke Otsuka, Kouki Uchinomiya, Yuki Yaguchi, and Atsushi Shibata

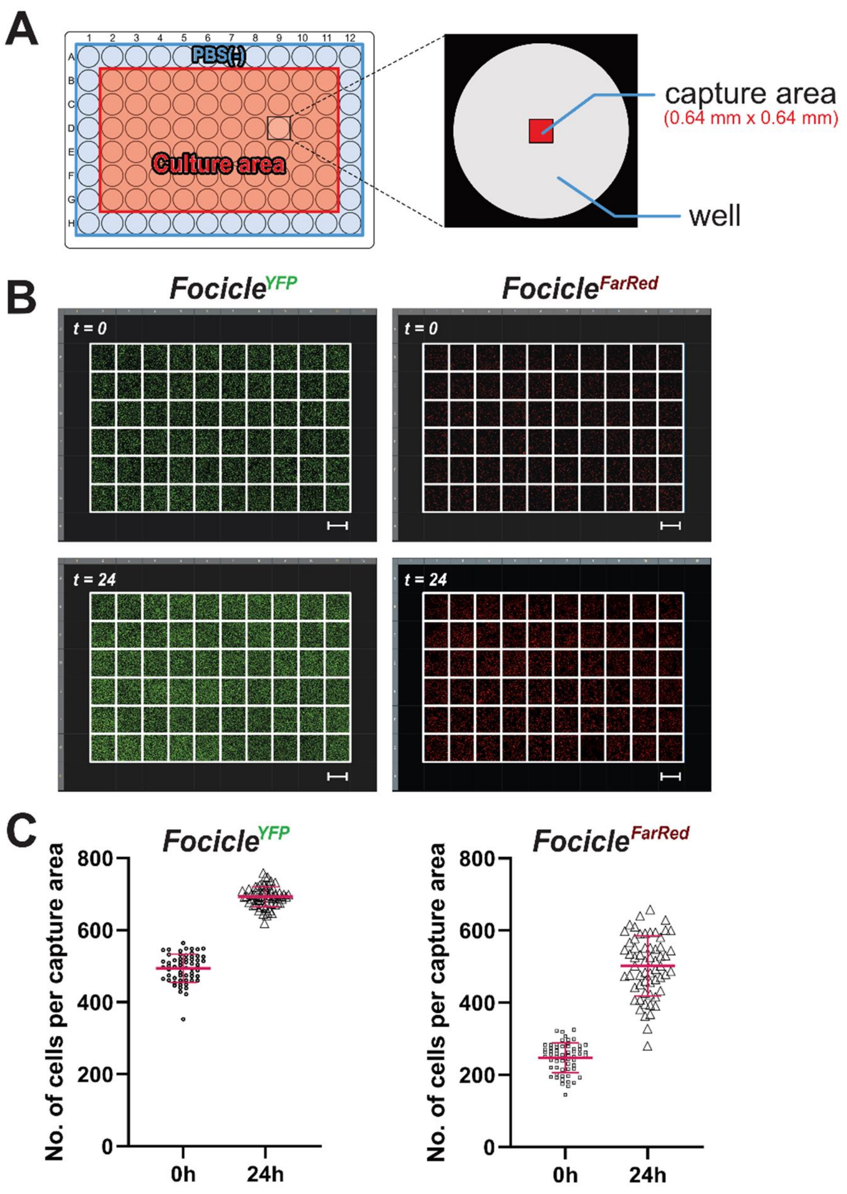

Figure S1. Experimental design of confocal live-cell imaging on 96-well format, Related to Figure 3. (A) Taking into account an optimal thermal distribution, we used centered 60 wells for live-cell imaging and peripheral 36 wells were filled with PBS(-) optimal thermal distribution. For time-lapse imaging, we took images from the center of the well (0.64 mm x 0.64 mm) every 1 h for 24 hrs. (B) A tiled image of YFP^{+} (Left) and FarRed^{+} (Right) cells dispensed in 96-well plate using an automated liquid dispensing workstation. Images were captured t=0h, and t=24h. In our setting, single loop took about 30 minutes for capturing 60 images. Scale bars, 500µm. (C) Number of cells per capture area (60 replicates) analyzed by a NIS-Elements software at t=0h and t=24h of YFP^{+} (Left) and FarRed^{+} (Right) cells. Solid magenta lines and error bars indicate their mean ± S.D.

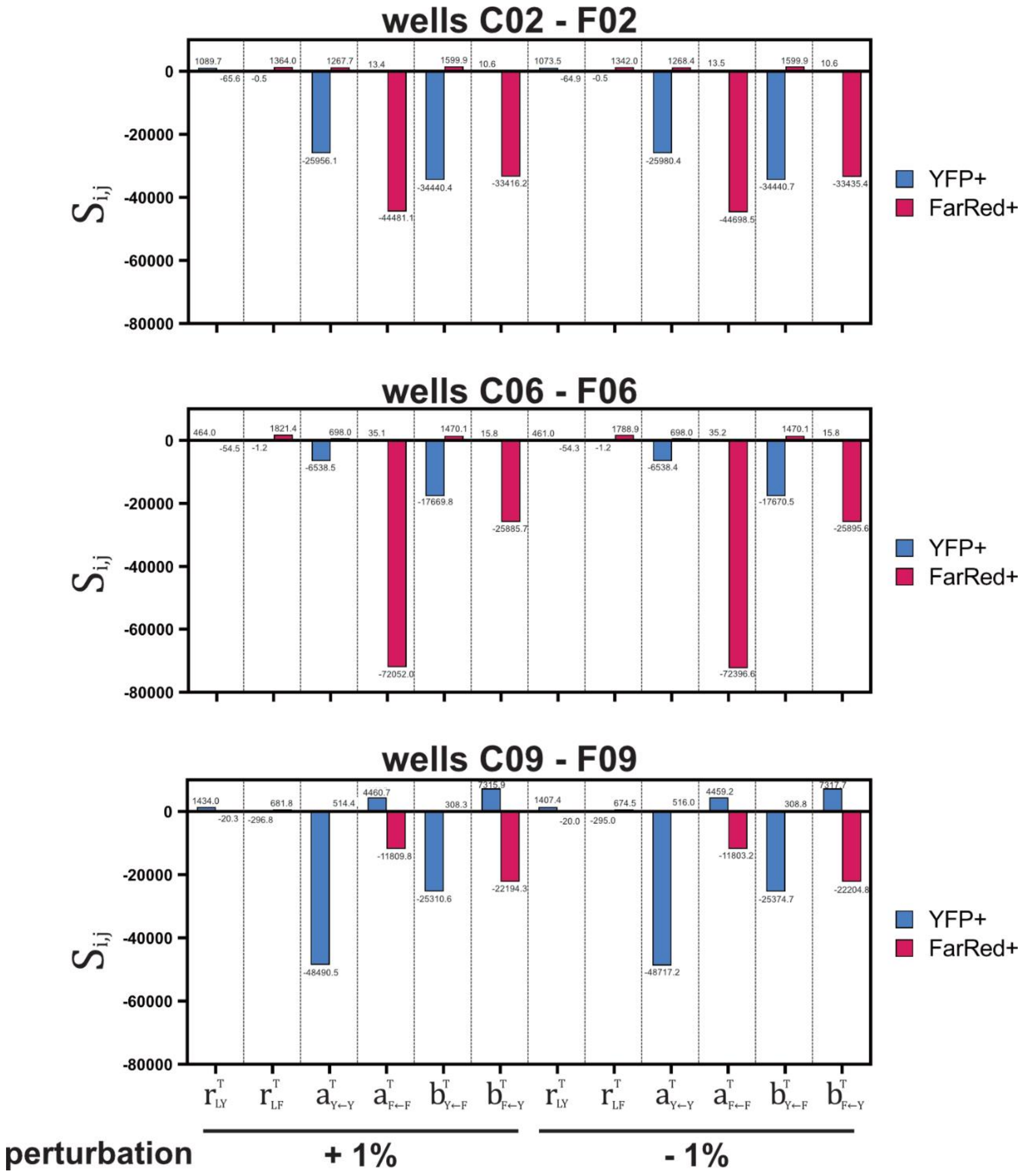

Figure S2. Parametric perturbation of YFP$^+$ and FarRed$^+$ cells 24 hours after the start of the co-culture, Related to Figure 3, Data S1.

The X-axis represents the parameters being perturbed, with a 1% increase (+) or decrease (-) in the estimated parameters. The Y-axis shows the sensitivity coefficient, representing the degree of change in the number of YFP$^+$ cells (blue bars) and FarRed$^+$ cells (magenta bars) at the 24-hour in response to each parameter variation. The specific values of each bar indicate the data of the sensitivity coefficient. Here, we exemplify three characteristic patterns of analysis: wells C02-F02 (top), wells C06-F06 (middle), and wells C09-F09 (bottom), shown in Figure 3C.

A

Abl1	*Ets1*	*Pten*
Akt1	*Ets2*	*Rb1*
Bmi1	*Fn1*	*Rbl1*
Calr	*Gsk3b*	*Rbl2*
Ccna2	*Hras1*	*Serpine1*
Ccnb1	*Id1*	*Sirt1*
Ccnd1	*Igf1r*	*Sod1*
Cd44	*Igfbp7*	*Sod2*
Cdc25c	*Ing1*	*Sparc*
Cdk2	*Irf3*	*Tbx3*
Cdk4	*Irf5*	*Terf2*
Cdk6	*Irf7*	*Tgfb1*
Cdkn1a	*Map2k1*	*Tgfb1i1*
Cdkn1b	*Map2k3*	*Thbs1*
Chek1	*Mapk14*	*Trp53*
Chek2	*Mdm2*	*Trp53bp1*
Cited2	*Morc3*	*Twist1*
Col1a1	*Myc*	*Vim*
Col3a1	*Nbn*	*Actb*
Creg1	*Nfkb1*	*B2m*
E2f1	*Pcna*	*Gapdh*
E2f3	*Pik3ca*	*Gusb*
Egr1	*Prkcd*	*Hsp90ab1*

B

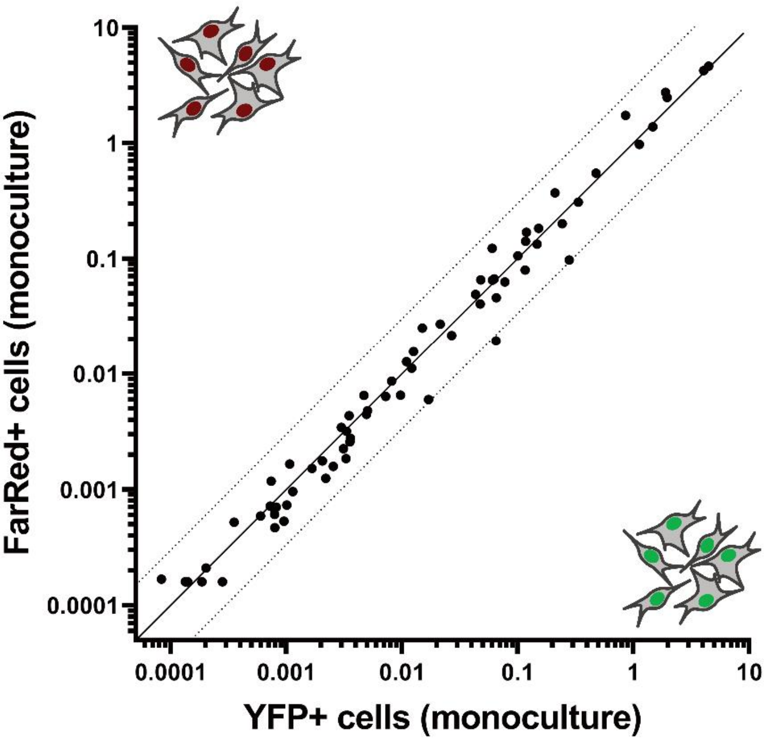

Figure S3. PCR array for comparison of two cell lines, Related to Figure 4.

(A) Gene list of RT^2 profiler PCR Array (#PAMM-050ZA, QIAGEN) for detecting an expression for mouse cellular senescence (Left). Total RNA isolated from YFP^+ and $FarRed^+$ monoculture and compared the relative expression for 69 genes (Right). (B) Fold change distribution of YFP^+ and $FarRed^+$ monocultured cells. Solid line shows fold change = 1 between YFP^+ and $FarRed^+$ cells. The dotted line shows fold change = 3 (upper) and 1/3 (lower).

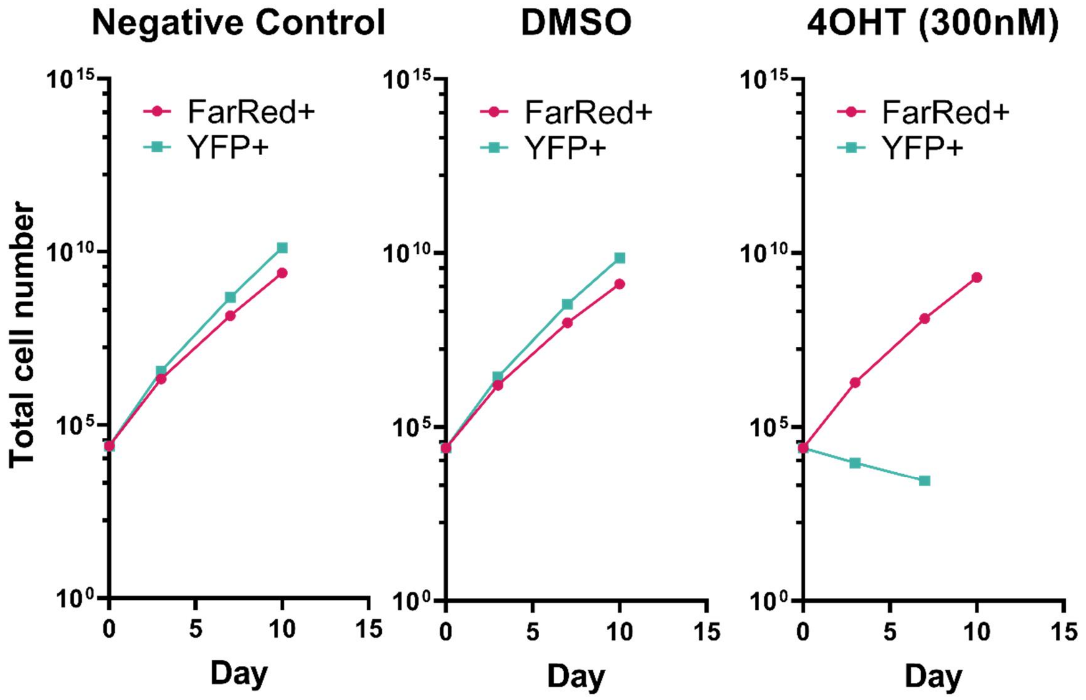

Figure S4. Cell growth after 300 nM 4OHT treatment, Related to Figure 4.

Cumulative cell number of mixed culture in negative control (Left), DMSO (Center), and 300 nM 4OHT treatment (Right). The cell number estimated based on the calculation of composition of YFP^+ and $FarRed^+$ cells using cell sorter and cell counter. YFP^+ cells were completely diminished at Day 10.

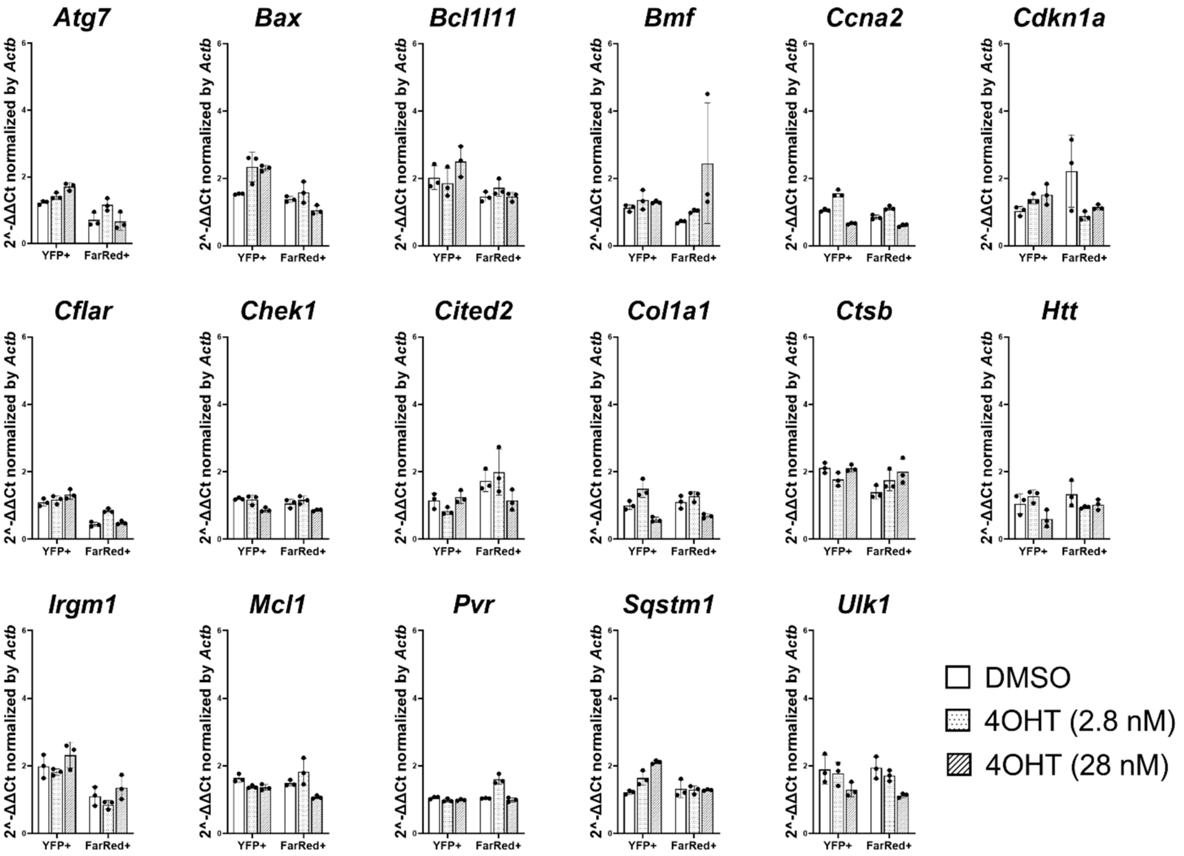

Figure S5. Genome stress array applied to YFP$^+$ and FarRed$^+$ cells isolated from mixed culture, Related to Figure 4.

Gene expressions of *Atg7, Bax, Bcl1l11, Bmf, Ccna2, Cdkn1a, Cflar, Chek1, Cited2, Col1a1, Ctsb, Htt, Irgm1, Mcl1, Pvr, Sqstm1,* and *Ulk1* were normalized by *Actb*. Total RNA was isolated from YFP$^+$/FarRed$^-$ or YFP$^-$/FarRed$^+$ fractions cultured with DMSO, 4OHT (2.8 nM), and 4OHT (28 nM). Bar graphs expressed mean ± S.D. of triplicate in one representative experiment. 2^{-ddCt} value was calculated with the baseline expression of the same fractions isolated from negative control.

Table S1: Fitting parameters for monoculture of YFP+ cells, Related to Figure 3.

Well	cell number (t=0)	cell number (t=24)	*r_Ex*	*N0_Ex*	*AIC_Ex*	*r_Logi*	*N0_L*	*AIC_Logi*	*a*	selected_model	Doubling Time (h)	a_Logi
B02	98	239	0.040	92.380	69.785	0.042	92.027	71.71	0.0000094	Exponential	18.7	
B03	102	277	0.045	95.232	102.438	0.057	92.022	102.77	0.0000632	Exponential	16.7	
B04	87	259	0.045	87.282	64.213	0.039	88.953	63.91	-0.0000395	Logistic		-0.0000395
B05	94	269	0.044	95.204	76.437	0.044	95.197	78.44	0.0000001	Exponential	15.8	
B06	74	276	0.057	68.707	79.022	0.047	71.263	77.74	-0.0000600	Logistic		-0.0000600
B07	64	220	0.048	70.755	98.267	0.043	71.763	100.06	-0.0000333	Exponential	13.5	
B08	83	295	0.056	80.281	101.024	0.060	79.023	102.71	0.0000225	Exponential	13.1	
B09	83	262	0.049	79.605	90.380	0.056	77.826	91.52	0.0000420	Exponential	14.5	
B10	102	333	0.054	93.297	84.417	0.060	91.358	85.00	0.0000273	Exponential	14.1	
B11	65	218	0.053	59.403	91.419	0.036	62.627	90.41	-0.0001256	Logistic		-0.0001256
C02	133	298	0.033	133.704	99.755	0.058	127.124	94.41	0.0001157	Logistic		0.0001157
C03	114	322	0.043	116.644	117.139	0.085	103.348	99.35	0.0001909	Logistic		0.0001909
C04	120	323	0.045	110.116	88.064	0.052	107.971	88.72	0.0000315	Exponential	16.8	
C05	102	363	0.049	111.350	108.242	0.061	107.122	107.89	0.0000543	Logistic		0.0000543
C06	122	380	0.047	119.848	120.521	0.060	115.211	120.91	0.0000552	Exponential	14.6	
C07	111	337	0.050	100.281	104.801	0.051	99.774	106.76	0.0000076	Exponential	15.0	
C08	85	325	0.052	93.741	97.125	0.064	90.145	96.21	0.0000554	Logistic		0.0000554
C09	140	396	0.044	140.144	94.406	0.057	135.071	89.98	0.0000484	Logistic		0.0000484
C10	116	368	0.049	113.375	120.519	0.031	119.400	119.24	-0.0000764	Logistic		-0.0000764
C11	105	379	0.052	103.949	118.952	0.055	103.015	120.88	0.0000118	Exponential	13.0	
D02	107	289	0.045	93.838	92.786	0.028	98.504	88.71	-0.0000981	Logistic		-0.0000981
D03	100	300	0.046	100.365	107.166	0.078	90.790	94.03	0.0001600	Logistic		0.0001600
D04	113	318	0.041	117.512	83.775	0.048	115.289	84.17	0.0000355	Exponential	16.1	
D05	93	333	0.050	100.428	116.489	0.067	94.914	115.50	0.0000822	Logistic		0.0000822
D06	91	259	0.046	92.732	97.741	0.076	84.326	80.28	0.0001637	Logistic		0.0001637
D07	128	350	0.049	105.667	110.149	0.037	109.756	109.97	-0.0000568	Logistic		-0.0000568
D08	116	365	0.046	118.961	108.242	0.064	112.875	105.30	0.0000742	Logistic		0.0000742
D09	93	264	0.043	93.881	99.123	0.064	88.599	95.98	0.0001188	Logistic		0.0001188
D10	91	353	0.057	88.564	112.194	0.052	90.416	113.76	-0.0000261	Exponential	12.3	
D11	97	340	0.055	88.113	91.695	0.038	93.783	81.72	-0.0000863	Logistic		-0.0000863
E02	96	237	0.035	103.972	105.565	0.085	92.543	85.33	0.0002909	Logistic		0.0002909
E03	109	264	0.036	110.179	86.937	0.047	107.587	87.02	0.0000572	Exponential	18.8	
E04	118	292	0.036	119.551	102.476	0.058	113.664	99.10	0.0001088	Logistic		0.0001088
E05	98	272	0.048	88.637	95.602	0.061	84.829	94.25	0.0000764	Logistic		0.0000764
E06	113	273	0.039	108.598	98.507	0.056	103.974	96.60	0.0000934	Logistic		0.0000934
E07	80	244	0.042	88.262	95.673	0.071	81.380	86.69	0.0001806	Logistic		0.0001806
E08	87	262	0.050	83.219	93.391	0.063	79.594	91.99	0.0000766	Logistic		0.0000766
E09	123	334	0.045	111.441	104.592	0.017	120.189	89.05	-0.0001330	Logistic		-0.0001330
E10	112	270	0.040	109.620	109.091	0.058	104.588	107.81	0.0000901	Logistic		0.0000901
E11	109	364	0.050	108.249	83.610	0.059	105.076	81.77	0.0000394	Logistic		0.0000394
F02	63	204	0.045	71.803	105.574	0.095	61.188	85.91	0.0003586	Logistic		0.0003586
F03	80	205	0.045	75.672	108.922	0.084	66.756	98.69	0.0002620	Logistic		0.0002620
F04	88	207	0.035	91.102	88.643	0.035	91.101	90.64	0.0000000	Exponential	19.4	
F05	83	258	0.047	80.320	79.790	0.026	85.310	67.65	-0.0001338	Logistic		-0.0001338
F06	79	208	0.038	86.121	101.737	0.083	76.840	86.86	0.0002923	Logistic		0.0002923
F07	86	206	0.041	78.561	100.997	0.058	74.998	100.91	0.0001202	Logistic		0.0001202
F08	100	302	0.046	104.078	105.167	0.074	95.394	93.53	0.0001335	Logistic		0.0001335
F09	99	272	0.042	96.691	92.728	0.033	98.822	93.64	-0.0000488	Exponential	16.5	
F10	89	250	0.046	84.628	77.616	0.055	82.440	77.42	0.0000537	Logistic		0.0000537
F11	96	353	0.055	94.352	73.993	0.060	92.862	74.71	0.0000197	Exponential	12.8	
G02	81	213	0.039	90.020	109.266	0.090	78.284	88.51	0.0003204	Logistic		0.0003204
G03	80	180	0.039	78.437	104.578	0.066	73.134	102.18	0.0001944	Logistic		0.0001944
G04	98	223	0.034	99.122	106.370	0.080	89.469	95.99	0.0002913	Logistic		0.0002913
G05	83	221	0.040	87.378	91.210	0.069	81.104	82.31	0.0001820	Logistic		0.0001820
G06	103	245	0.033	106.800	104.127	0.033	106.798	106.13	0.0000000	Exponential	19.2	
G07	95	229	0.038	94.918	94.812	0.061	89.663	90.90	0.0001443	Logistic		0.0001443
G08	81	262	0.053	76.678	81.888	0.053	76.678	83.89	0.0000000	Exponential	14.2	
G09	102	264	0.045	88.402	104.669	0.012	96.371	92.74	-0.0001975	Logistic		-0.0001975
G10	62	189	0.048	64.973	98.118	0.085	57.369	86.07	0.0002787	Logistic		0.0002787
G11	96	333	0.051	92.262	105.158	0.049	92.847	107.11	-0.0000098	Exponential	13.4	
Median	*96.5*	*272*	*0.045*	*93.859*		*0.058*	*92.025*		*0.0000540*	20		*0.0000917*
SD	*17.1*	*55.5*	*0.0*	*16.2*		*0.0*	*16.3*		*0.000121*	40		*0.000140*
CV	*0.18*	*0.20*	*0.14*	*0.17*		*0.31*	*0.18*		*2.23*			*1.52*

r_Ex intrinsic natural growth rate in an exponential growth (eq 1)

N0_Ex number of initial cells per image estimated by an exponential model

AIC_Ex Akaike information criterion estimated by an exponential model

r_Logi intrinsic natural growth rate in a logistic growth (eq 2)

N0_L number of initial cells per image estimated by a logistic model

AIC_Logi Akaike information criterion estimated by a logistic model

a intraspecific competition coefficient

Table S2: Fitting parameters for monoculture of FarRed$^+$ cells, Related to Figure 3.

Well	cell number (t=0)	cell number (t=24)	*r_Ex*	*N0_Ex*	*AIC_Ex*	*r_Logi*	*N0_L*	*AIC_Logi*	*a*	selected_model	Doubling Time (h)	a_Logi
B02	76	202	0.039	79.41	79.76	0.056	76.161	77.66	0.0001214	Logistic	-	0.0001214
B03	78	186	0.038	75.83	104.57	0.078	68.515	98.71	0.0002978	Logistic	-	0.0002978
B04	75	212	0.044	76.84	87.41	0.054	74.671	87.94	0.0000670	Exponential	16.0	-
B05	78	205	0.044	71.33	79.17	0.042	71.791	81.08	-0.0000170	Exponential	17.2	-
B06	61	166	0.044	59.81	92.21	0.056	57.844	93.25	0.0000996	Exponential	16.6	-
B07	65	149	0.036	61.97	75.40	0.008	65.715	68.74	-0.0002740	Logistic	-	-0.0002740
B08	68	187	0.041	68.48	66.22	0.025	71.377	61.27	-0.0001361	Logistic	-	-0.0001361
B09	36	135	0.056	34.36	48.75	0.047	35.489	48.68	-0.0001125	Logistic	-	-0.0001125
B10	48	162	0.047	51.00	79.01	0.047	50.997	81.01	0.0000004	Exponential	13.7	-
B11	54	190	0.056	52.29	81.35	0.062	50.973	82.60	0.0000541	Exponential	13.2	-
C02	52	166	0.045	57.59	87.01	0.088	50.408	68.24	0.0003767	Logistic	-	0.0003767
C03	59	186	0.051	55.24	72.13	0.058	53.995	73.23	0.0000566	Exponential	14.5	-
C04	90	193	0.036	81.46	82.28	0.021	84.182	81.29	-0.0001137	Logistic	-	-0.0001137
C05	71	190	0.039	74.67	87.01	0.009	79.708	78.92	-0.0002293	Logistic	-	-0.0002293
C06	75	168	0.036	72.75	63.20	0.034	73.087	65.12	-0.0000175	Exponential	20.6	-
C07	75	186	0.044	69.12	91.88	0.037	70.414	93.43	-0.0000511	Exponential	18.3	-
C08	65	170	0.036	69.83	77.37	0.057	66.708	75.44	0.0001739	Logistic	-	0.0001739
C09	75	177	0.039	67.15	92.68	-0.001	73.241	82.42	-0.0003450	Logistic	-	-0.0003450
C10	59	189	0.044	62.47	86.21	0.054	60.760	87.39	0.0000851	Exponential	14.3	-
C11	63	195	0.047	60.34	79.73	0.015	65.908	61.38	-0.0002679	Logistic	-	-0.0002679
D02	89	227	0.041	87.40	77.14	0.039	87.820	79.05	-0.0000119	Exponential	17.8	-
D03	69	154	0.034	66.85	59.47	0.013	69.668	52.75	-0.0001978	Logistic	-	-0.0001978
D04	63	151	0.035	68.40	70.32	0.058	65.103	65.94	0.0001992	Logistic	-	0.0001992
D05	84	188	0.036	80.67	80.81	0.030	81.772	82.34	-0.0000473	Exponential	20.6	-
D06	72	177	0.037	72.53	79.29	0.020	75.207	78.17	-0.0001416	Logistic	-	-0.0001416
D07	66	167	0.041	61.56	91.40	0.003	67.271	81.16	-0.0003490	Logistic	-	-0.0003490
D08	113	403	0.049	130.20	122.47	0.083	115.882	101.72	0.0001228	Logistic	-	0.0001228
D09	74	205	0.046	68.85	101.59	0.019	74.224	97.10	-0.0002016	Logistic	-	-0.0002016
D10	48	155	0.052	44.83	77.68	0.031	48.159	73.21	-0.0002220	Logistic	-	-0.0002220
D11	59	190	0.055	52.76	76.18	0.066	50.651	75.81	0.0000875	Logistic	-	0.0000875
E02	93	206	0.038	81.63	103.18	0.025	84.029	104.20	-0.0000897	Exponential	20.9	-
E03	68	161	0.037	69.78	91.36	0.054	67.045	91.42	0.0001402	Exponential	19.3	-
E04	68	170	0.039	67.93	97.02	0.029	69.564	98.44	-0.0000843	Exponential	18.2	-
E05	81	199	0.039	74.56	67.07	0.022	77.678	61.52	-0.0001369	Logistic	-	-0.0001369
E06	67	170	0.038	62.93	95.65	-0.026	71.409	67.72	-0.0006133	Logistic	-	-0.0006133
E07	67	166	0.036	70.13	74.96	0.036	70.128	76.96	-0.0000002	Exponential	18.3	-
E08	65	182	0.043	65.86	83.55	0.058	63.068	82.71	0.0001210	Logistic	-	0.0001210
E09	54	151	0.040	59.45	88.48	0.024	61.899	88.50	-0.0001567	Exponential	16.2	-
E10	53	142	0.044	51.58	88.55	0.045	51.380	90.54	0.0000138	Exponential	16.9	-
E11	53	172	0.055	48.68	85.13	0.046	50.387	85.98	-0.0000841	Exponential	14.1	-
F02	84	180	0.035	74.89	89.76	0.005	79.361	85.44	-0.0002484	Logistic	-	-0.0002484
F03	58	153	0.037	62.15	87.91	0.017	64.788	87.67	-0.0001901	Logistic	-	-0.0001901
F04	44	120	0.037	52.03	81.72	0.086	46.163	69.85	0.0005519	Logistic	-	0.0005519
F05	61	134	0.033	58.78	76.04	0.002	62.146	72.25	-0.0003408	Logistic	-	-0.0003408
F06	71	156	0.032	74.08	71.71	0.049	71.588	70.44	0.0001514	Logistic	-	0.0001514
F07	37	100	0.046	35.81	75.30	0.085	31.598	67.57	0.0005429	Logistic	-	0.0005429
F08	75	182	0.043	68.61	91.02	0.041	68.988	92.98	-0.0000159	Exponential	18.8	-
F09	58	165	0.041	61.55	81.66	0.045	60.933	83.52	0.0000352	Exponential	15.9	-
F10	63	178	0.045	60.89	86.67	0.072	56.037	80.68	0.0002285	Logistic	-	0.0002285
F11	40	143	0.061	33.53	69.11	0.046	35.637	67.49	-0.0001703	Logistic	-	-0.0001703
G02	89	216	0.037	90.11	109.91	0.087	79.735	99.00	0.0003227	Logistic	-	0.0003227
G03	65	172	0.037	69.16	74.20	0.046	67.818	75.37	0.0000734	Exponential	17.1	-
G04	86	191	0.035	85.11	87.32	0.027	86.590	88.64	-0.0000591	Exponential	20.8	-
G05	65	149	0.034	66.72	77.87	0.069	61.882	70.04	0.0003226	Logistic	-	0.0003226
G06	72	161	0.030	83.33	86.18	0.063	78.430	81.03	0.0002569	Logistic	-	0.0002569
G07	61	181	0.040	69.54	73.16	0.063	65.472	66.06	0.0001940	Logistic	-	0.0001940
G08	67	193	0.046	65.89	67.31	0.045	66.001	69.31	-0.0000044	Exponential	15.7	-
G09	60	172	0.049	58.39	100.83	0.098	48.928	79.90	0.0004026	Logistic	-	0.0004026
G10	58	187	0.048	58.05	47.13	0.036	60.150	41.01	-0.0001037	Logistic	-	-0.0001037
G11	77	243	0.046	84.58	91.99	0.074	78.153	84.76	0.0001728	Logistic	-	0.0001728
Median	*67*	*177*	*0.041*	*67.54*	*81.69*	*0.05*	*67.16*	*80.29*	*-0.0000081*	23		
SD	*14.1*	*38.6*	*0.0*	*14.9*	*13.3*	*0.0*	*14.1*	*12.9*	*0.0002226*	37		
CV	*0.21*	*0.22*	*0.16*	*0.22*	*0.16*	*0.57*	*0.21*	*0.16*	*-27.33*			

r_Ex intrinsic natural growth rate in an expnential growth (eq 1)

N0_Ex number of initial cells per image estimated by an exponential model

AIC_Ex Akaike information criterion estimated by an exponential model

r_Logi intrinsic natural growth rate in a logistic growth (eq 2)

N0_L number of initial cells per image estimated by a logistic model

AIC_Logi Akaike information criterion estimated by a logistic model

a intraspecific competition coefficient

Data S1: Parametric Sensitivity Analysis. Related to Figure 3

To examine which parameter changes are critical for the estimation of cell numbers in the mixed culture (Lotka-Volterra competition equations) used in this study, we conducted a parameter sensitivity analysis. The equations analyzed are those described in Equations (3a) and (3b) in the main text, as shown below.

$$\frac{dN_F(t)}{dt} = r_{LF}^T N_F(t) - a_{F\leftarrow F}^T N_F(t)^2 - b_{F\leftarrow Y}^T N_F(t) N_Y(t) \qquad (3a)$$

$$\frac{dN_Y(t)}{dt} = r_{LY}^T N_Y(t) - a_{Y\leftarrow Y}^T N_Y(t)^2 - b_{Y\leftarrow F}^T N_Y(t) N_F(t) \qquad (3b)$$

The equations $\frac{dN_F(t)}{dt}$ and $\frac{dN_Y(t)}{dt}$ shown in Equation (3a, 3b) each exhibit logistic growth when $N_Y(t) = 0$ and $N_F(t) = 0$, respectively.

In this study, we determined the parametric perturbation according to Equation (5) as presented by Perumal & Gunawan (2011)[S1].

$$S_{i,j} = \frac{\partial x_i}{\partial p_j} \qquad (5)$$

where $S_{i,j}$ is referred to as the sensitivity coefficient, indicating how the state of the system x_i when the parameter p_j is slightly altered. This has been adapted to the Lotka-Volterra competition equations presented in this paper. For example, the change in the number of FarRed cells at the 24-hour mark when the intrinsic growth rate r_{LF} of the FarRed cells is varied can be expressed by the following equation:

$$\frac{N_F(24) - \widehat{N}_F(24)}{r_{LF} - \hat{r}_{LF}}$$

where, $N_F(24)$ represents the number of FarRed cells at the 24-hour mark using the estimated parameters, while $\widehat{N}_F(24)$ represents the number of FarRed cells at the 24-hour mark when the estimated parameter r_{LF} is altered. $\hat{r}_{LF}$ is a value obtained by varying of r_{LF} by a certain amount. In this case, the rate of change was set to a 1% increase or decrease. Based on these assumptions, the results of the parametric perturbation for each well are presented in Supplemental Fig. 1.

When all the intrinsic growth rates and intraspecific competition coefficients included in the Equation (3a/3b) were altered, almost all wells exhibited the same trend; specifically, the impact of changes in the intrinsic growth rates was small, whereas the impact of changes in the intraspecific competition coefficients was observed to be significant. In the following, we interpret the parametric perturbation when the parameters

are increased. Focusing on intraspecific interactions, when the influence of YFP cells on themselves $\mathrm{a}_{Y \leftarrow Y}^{\mathrm{T}}$ increased, the parametric perturbation for YFP became significantly negative, while that for FarRed became slightly positive. This indicates that an increase in $\mathrm{a}_{Y \leftarrow Y}^{\mathrm{T}}$ leads to a decrease in YFP cells and a slight increase in FarRed cells. Conversely, when the influence of FarRed cells on themselves $\mathrm{a}_{F \leftarrow F}^{\mathrm{T}}$ increased, FarRed cells decreased, while YFP cells slightly increased. Focusing on the interactions between different cell types, when the influence of FarRed cells on YFP cells $b_{Y \leftarrow F}^{\mathrm{T}}$ increased, YFP cells decreased, while FarRed cells slightly increased. Conversely, when the influence of YFP cells on FarRed cells $b_{F \leftarrow Y}^{\mathrm{T}}$ increased, FarRed cells decreased, while YFP cells slightly increased. These results suggest that changes in cell numbers are more strongly dependent on cell-cell interactions than on the intrinsic growth potential unaffected by interactions.

REFERENCES

[S1]Perumal, T.M. and Gunawan, R. (2011) Understanding dynamics using sensitivity analysis: caveat and solution. BMC Syst. Biol. 5, 41. 10.1186/1752-0509-5-41.
